# Decoding Human Placental Cellular and Molecular Responses to Obesity and Fetal Growth

**DOI:** 10.1002/advs.202509691

**Published:** 2026-01-20

**Authors:** Hong Jiang, Emilie Derisoud, Denise Parreira, Nayere Taebnia, Paulo R. Jannig, Reza Zandi Shafagh, Allan Zhao, Congru Li, Macarena Ortiz, Manuel Alejandro Maliqueo, Elisabet Stener‐Victorin, Volker M. Lauschke, Qiaolin Deng

**Affiliations:** ^1^ Karolinska Institutet Department of Physiology and Pharmacology Stockholm Sweden; ^2^ Stockholm University Department of Molecular Biosciences The Wenner‐Gren Institute Stockholm Sweden; ^3^ Center For Molecular Medicine Karolinska Institutet and University Hospital Stockholm Sweden; ^4^ Dr Margarete Fischer‐Bosch Institute of Clinical Pharmacology Stuttgart Germany; ^5^ University of Chile Departamento De Medicina Interna Santiago Chile; ^6^ Division of Micro‐ and Nanosystems KTH Royal Institute of Technology Stockholm Sweden; ^7^ Department of Pharmacy the Second Xiangya Hospital Central South University Changsha China

**Keywords:** birth weight, maternal obesity, microfluidic co‐culture, placental transcriptomics, single‐nucleus RNA sequencing

## Abstract

Maternal obesity increases the risks of large‐for‐gestational‐age (LGA) births and subsequent cardiometabolic disorders in offspring. To identify placental signatures associated with these outcomes, we performed single‐nucleus RNA sequencing on placentas from women with obesity delivering appropriate‐for‐gestational‐age or LGA infants, compared to normal‐weight controls. In maternal obesity, regardless of fetal growth, syncytiotrophoblasts showed upregulated hypoxia and TNF‐α signaling, while cytotrophoblasts exhibited downregulated receptor tyrosine kinase signaling. However, villous non‐trophoblasts displayed upregulated TNF‐α signaling and inflammatory responses only in LGA placentas. Notably, Hofbauer cells in LGA placentas presented transcriptional alterations in immunometabolism‐related genes and displayed elevated *SPP1* expression, which potentially acts as a ligand for other placental cell types. We modeled key aspects of syncytiotrophoblast responses to adipose tissue using a customized microfluidic organoids‐on‐a‐chip co‐culture system. These findings revealed gene expression patterns of placental cells to maternal obesity that are shared or different between O‐A and O‐L, highlighting pathways for future mechanistic investigation.

## Graphical abstract

Women with obesity often deliver large‐for‐gestational‐age (LGA) infants. Single‐nucleus RNA sequencing of term placenta reveals that hypoxia and TNF‐α signaling in syncytiotrophoblasts are featured in maternal obesity, but inflammatory signatures in Hofbauer cells and response to lipid or carbohydrate metabolism in fibroblasts are specific to LGA. A microfluidic co‐culture models trophoblast responses to adipose tissue providing a minimalistic approach to study the maternal obesity on placentas.

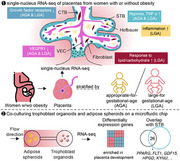

## 1 Introduction

The global prevalence of obesity among women of reproductive age reached 18.5% in 2022, making maternal obesity an increasing concern for women's health [1]. Maternal obesity is manifested by chronic low‐grade inflammation, hyperinsulinemia, and dysregulated lipid metabolism [2]. Consequently, these systemic changes collectively impact the intrauterine environment and lead to immediate and long‐term health consequences for both mothers and their offspring, including a greater risk of developing cardiometabolic diseases and type 2 diabetes [3, 4, 5]. Among pregnancy complications, delivery of large‐for‐gestational‐age (LGA) infants (birth weight > 90th percentile) [6] is particularly prevalent, occurring in 13.41%–38.3% of pregnancies affected by obesity [7, 8, 9, 10]. LGA itself is not only a pregnancy complication but also a critical risk factor for further adverse outcomes, including birth trauma [11], childhood obesity [12], cardiometabolic and neurological disorders in early adulthood [13]. Therefore, preventing LGA may serve as an effective strategy to mitigate the cascade of associated complications.

Maternal obesity is often associated with excessive gestational weight gain [14], which subsequently contributes to a higher risk of LGA infants due to excess nutrient supply [15]. However, the UK UPBEAT study, a randomized controlled trial testing a tailored complex lifestyle intervention (diet and physical activity) in pregnant women with obesity, found that a reduction in gestational weight gain did not have a significant effect on the outcomes of gestational diabetes and LGA infants [16]. Also, the EMPOWaR study found that pharmacological use of metformin in pregnant women with obesity had no significant effect on reducing birth weight percentiles or preventing adverse neonatal outcomes despite clearly improving maternal metabolic health [17]. These studies collectively identify potential additional factors contributing to fetal growth and the shortcomings of current interventions in preventing LGA infants, underscoring the need for a deeper understanding of the underlying mechanisms.

Intriguingly, not all fetuses exposed to maternal obesity exhibit excessive growth and develop LGA, with some still following a normative growth trajectory resulting in appropriate‐for‐gestational‐age (AGA). This variability may reflect an inherent adaptive mechanism in utero that could be harnessed for future LGA management. The placenta is the central structure at the maternal–fetal interface, regulating the quantity and quality of nutrient supply from the maternal circulation by adjusting its transport capacity and nutrient uptake [18]. Additionally, it dynamically modulates growth factors and hormonal secretion [19] and adjusts inflammation levels [20] to align with the growth requirements of the fetus. These activities are intertwined and could serve as the link between maternal obesity and divergent fetal growth trajectories. For example, elevated maternal insulin levels in obesity activate mTOR signaling as well as glucose and amino acid transport in the placenta, thus contributing to accelerated fetal growth in some women with obesity [21]. Circulating pro‐inflammatory cytokines in obesity, such as IL‐6, tumor necrosis factor‐α (TNF‐α), and leptin, are found to promote lipid storage in the placenta [20] and enhance amino acid and lipid transporters in trophoblast. Moreover, secretion of placental lactogen and prolactin is correlated with pre‐pregnancy BMI and fetal growth [22] highlighting the placenta's active role in modulating the effects of obesity on fetal growth [23].

Current understanding of the placental response to maternal obesity largely relies on gene expression analyses conducted at the whole‐tissue level or in cell cultures [24, 25, 26, 27]. These approaches are often confounded by tissue complexity or lack of physiological relevance in culture conditions. A recent single‐nucleus study of placentas from pregnancies with maternal obesity reported activation of the hypoxia pathway across multiple cell types, with analysis and validation centered on EVTs. However, placentas were not stratified by fetal growth outcomes (AGA or LGA) [28]. Identifying molecular signatures in the placenta associated with AGA and LGA infants at a single‐cell level may provide valuable insights into cell‐type‐specific mechanisms driving distinct growth patterns and inform potential targeted therapeutic strategies. Here, we performed single‐nucleus RNA sequencing (snRNA‐seq) on term placentas collected in a Chilean cohort study. The cohort excluded women with pregnancy complications such as hypertension, preeclampsia, thyroid disorders, gestational diabetes, preterm delivery, fetal malformations or chromosomal aberrations, as well as those with pregestational diabetes, polycystic ovary syndrome, infertility, or assisted fertilization (IVF or ICSI) [29]. Among women with obesity, no differences were reported in circulating glucose, lipids, or dietary intake of major fatty acids compared to controls [29]. Thus, this cohort provided a unique opportunity to examine obesity‐associated placental transcriptional changes in the absence of confounding metabolic or obstetric complications, aside from more frequent delivery of LGA infants. We divided the placental samples and analyzed three groups: normal BMI with AGA infants (Control), maternal obesity with AGA infants (O‐A), and maternal obesity with LGA infants (O‐L). Cell‐type‐specific transcriptomic profiles and pathways were identified as either shared or different in O‐A and O‐L groups, suggesting maternal obesity‐associated patterns that are shared or different between O‐A and O‐L. The intercellular ligand–receptor communication network revealed the major cell types mediating these shared or different responses. Microfluidic co‐culture of adipose spheroids and trophoblast organoids partially recapitulates transcriptional changes of syncytiotrophoblast in maternal obesity, providing an in vitro model with mechanistic insights to study the effects of maternal conditions on human placentas in the future.

## 2 Results

### 2.1 Cell Types in Placentas Stratified by Maternal Obesity and Fetal Growth

**FIGURE 1:**
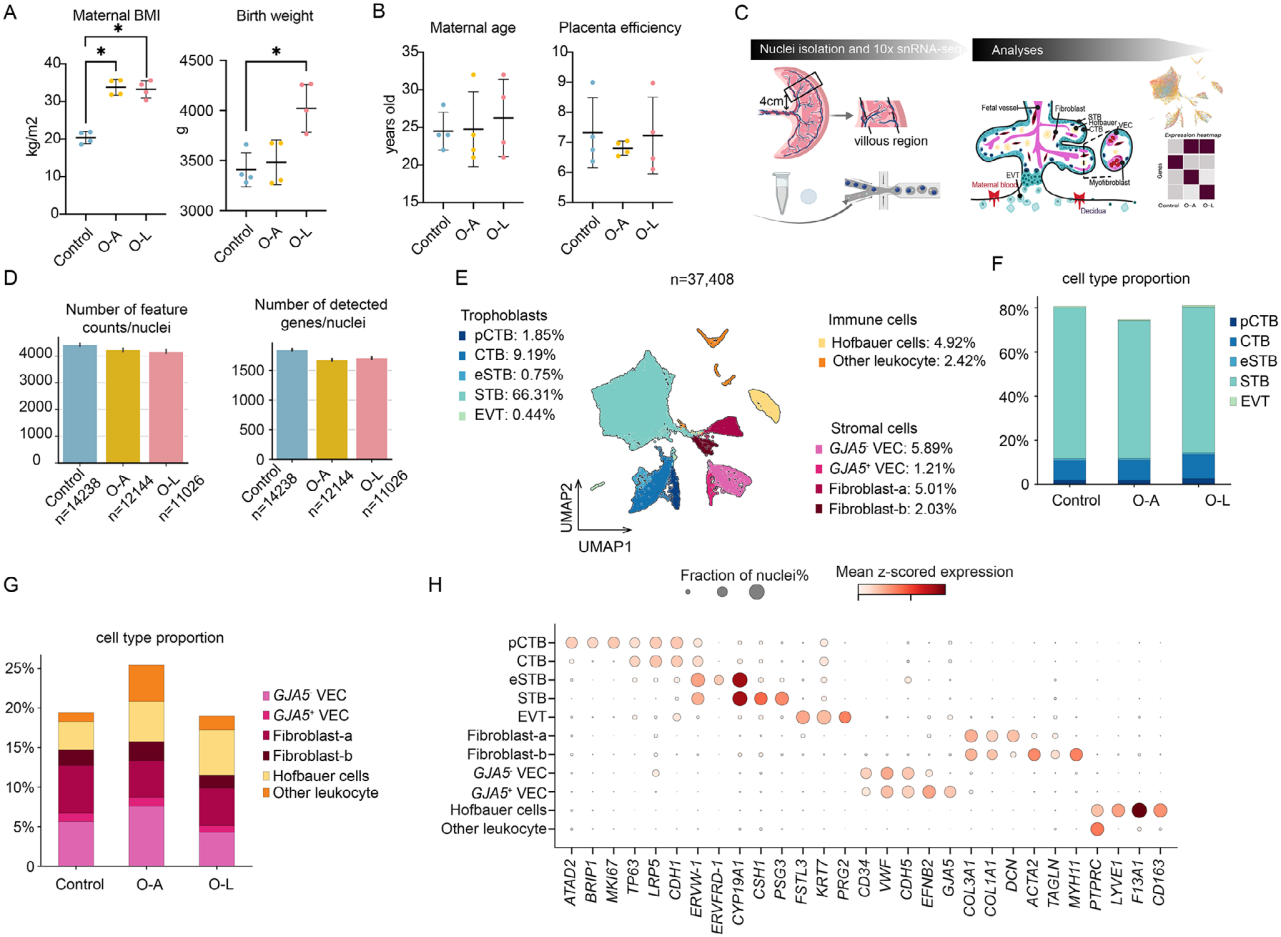
snRNA‐seq of human placentas from normal‐weight mothers and mothers with obesity stratified by fetal birth weight. (A) Maternal BMI (body mass index) and birth weight of samples for snRNA‐seq from each group. (B) Maternal age and placental efficiency of samples for snRNA‐seq from each group. (C) Experimental procedures. Created with BioRender.com and modified with Adobe Illustrator. (D) Number of feature counts per nucleus and number of detected genes per nucleus. (E) Two‐dimensional UMAP (Uniform manifold approximation and projection) of 37 408 nuclei profiles (dots) from all donors (*n *= 12), colored by cell type. (F) Stacked barplot showing trophoblast cell proportion in each group. (G) Stacked barplot showing the proportion of non‐trophoblast cell types in each group. (H) Mean expression (dot color, gene expression scaled to unit variance) and fraction of expressing nuclei (dot size) of marker genes for each of the 11 major cell types. Controls: normal‐weight women (*n *= 4); O‐A: Mothers with obesity with appropriate‐for‐gestational‐age baby (birth weight within the 25th and 90th percentile of WHO growth curves, *n* = 4); O‐L: Mothers with obesity with large‐for‐gestational‐age baby (birth weight > 90th percentile of WHO growth curves, *n* = 4); CTB: cytotrophoblasts; VEC: vascular endothelial cells; EVT: extravillous trophoblasts; pCTB: proliferative CTB; STB: syncytiotrophoblasts; eSTB: early syncytiotrophoblasts. *n* = 4 for each group in (A, B), Kruskal–Wallis test followed by Dunn's multiple comparisons test; error bars indicate mean ± sd.

To investigate placental gene expression that showed maternal obesity‐associated patterns shared or different between O‐A and O‐L, we selected 12 samples from a birth cohort [29] with representative maternal BMI and birth weight for snRNA‐seq (Figure 1A; Figure S1A). Both sexes were included in the control, O‐A, and O‐L groups, and all samples were obtained from vaginal delivery. The two maternal obesity groups (O‐A and O‐L) showed no significant differences in maternal age, placental weight, and placental efficiency compared with controls (Figure 1A,B). We dissected the placental villous region, dissociated tissue into single nuclei, and prepared libraries using 10x Chromium technology (Figure 1C). Given the limited number of placentas analyzed (*n* = 4 per group), we aimed to provide observation about the gene expression patterns.

We obtained an average of 108 323 reads per sample with a median of 1395 genes per cell (Table S1). Nuclei (37 408) were retained for downstream analyses, yielding comparable total counts and gene number per cell across all groups (Figure 1D). The transcriptomic profiles of nuclei did not cluster by individual (Figure S1B). We did not observe any pronounced sex‐specific clustering of cells (Figure S1C). Using canonical marker genes for cell types [30], we annotated 11 placental cell types: five types of trophoblasts, two of endothelial cell states, two fibroblast types, Hofbauer cells, and other leukocytes (Figure 1E). Cell type proportions, including various trophoblast and villous non‐trophoblast populations, showed no significant differences among the three groups (Figure 1F,G, Table S1).

Trophoblasts constituted the majority of cells, primarily syncytiotrophoblast (STB) and villous cytotrophoblasts (CTBs). Proliferative CTBs (pCTBs) were distinguished by cell cycle markers (Figure 1H; Figure S1D). Co‐expression of *ERVW‐1* (Syncytin‐1) and *ERVFRD‐1* (Syncytin‐2) identified early STB (eSTB), consistent with previous reports [31]. Mature STB shared expression of *ERVW‐*1 and *CYP19A1* with eSTB but uniquely expressed *CSH1* and *PSG3* (Figure 1H). EVTs comprised only 0.5% of all nuclei, with fewer than 100 cells per group, reflecting our villous‐focused sampling approach (Table S1). Among other villous non‐trophoblast cells, we detected two fibroblast‐like populations (fibroblast‐a and b). Both expressed extracellular matrix genes, including collagens (*COL3A1* and *COL1A1*), while fibroblast‐b resembles myofibroblasts/pericytes through concurrent expression of smooth muscle markers (*ACTA2*, *TAGLN*, *MYH11)* (Figure 1H). Vascular endothelial cells (VECs) were divided into *GJA5^−^* and a small subset of *GJA5* ^+^ subsets (Figure 1H; Figure S1E). *GJA5* encodes a gap junction protein facilitating direct intercellular communication. Among the immune cells, we primarily captured Hofbauer cells, specialized macrophages residing within the chorionic villi (Figure 1H; Figure S1F). Additional *PTPRC+* nuclei indicated other leukocyte populations (Figure 1H, Table S1). However, these cells were excluded from subsequent analyses due to the limited numbers and peripheral relevance to our study objectives (Figure S1G).

Next, we focused on the major villous cell types (STB, CTB, VEC, fibroblast‐a, and Hofbauer cells) to elucidate their molecular responses to obesity and fetal growth regulation. We performed differential gene expression analyses comparing each obesity group to the control group, including sex as a covariate in the model. This approach ensured that the gene expression differences identified were attributable to the obesity group rather than to sex. To support these observations, we introduced a continuous “C‐score” that integrates fold‐change magnitude and significance from the two comparisons. Genes with positive C‐scores had concordant up‐ or down‐regulation in both O‐A and O‐L (i.e., shared maternal obesity‐associated patterns), whereas negative C‐scores identified different maternal obesity‐associated patterns between O‐A and O‐L (Figure S1H). This ranking supplemented our analysis by confirming which genes/pathways showed shared versus different patterns (Table S2, Methods).

### 2.2 Identification of STB Subpopulations Along the Maturation States

**FIGURE 2:**
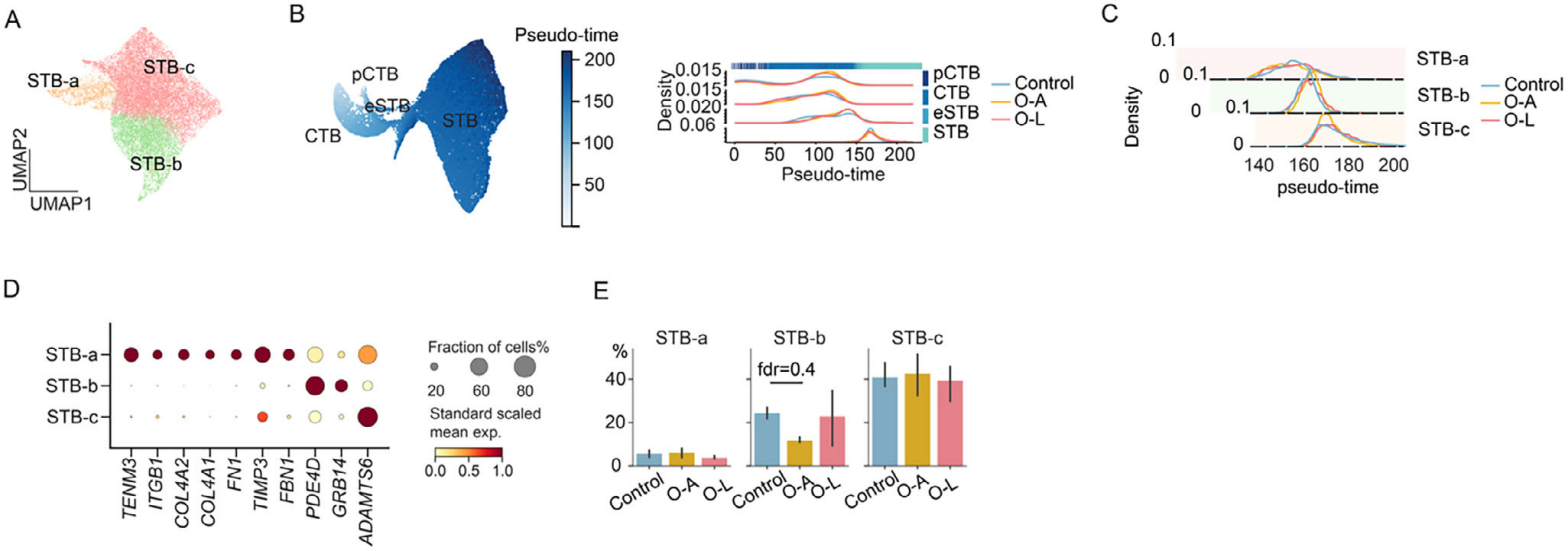
Syncytiotrophoblast (STB) nuclei substates along pseudotime. (A) Two‐dimensional Uniform manifold approximation and projection (UMAP) representation of STB colored by STB clusters. (B) UMAP representation of villous trophoblasts (CTB, cytotrophoblast; pCTB, proliferative CTB; STB, syncytiotrophoblasts; eSTB, early STB) colored by inferred pseudo time (left). Pseudotime value visualization across trophoblast cell types and groups (right). (C) Pseudotime value visualization across STB states and groups. (D) Dotplot of genes specifically expressed in STB‐a, ‐b, or ‐c state. Dot color indicates the standard‐scaled mean expression level of the gene. (E) Proportion of STB states across groups. Bar height represents the mean, and the error bar represents a 50% interval. Data were compared by a Bayesian model.

The STB layer forms through CTB fusion, requiring extensive cytoskeletal and plasma membrane remodeling. We captured 24 478 STB nuclei and studied subpopulations of STB. Re‐clustering STB nuclei revealed three substates (STB‐a, ‐b, and ‐c) (Figure 2A). To characterize their maturation trajectory, we incorporated CTBs and eSTB as early differentiation timepoints for pseudo‐time analysis (Figure 2B; Figure S2A). The three STB substates aligned along the pseudo‐time axis with varying distributions (Figure 2C). Sub‐state specific markers highlighted biological distinctions between these states (Figure 2D; Figure S2B). STB‐a represented an earlier stage characterized by *TENM3* expression (Figure 2D; Figure S2B). *TENM3* encodes a teneurin transmembrane protein promoting homophilic cellular adhesion [32]. *TENM3* ^+^ STB's localization in the STB layer was demonstrated by in situ hybridization [33]. This state was further characterized by genes regulating the cytoskeleton, tight junctions, and extracellular matrix (*ITGB1*, *COL4A1, COL4A2*, *FN1*, and *TIMP3*) (Figure 2D). STB‐b specifically expressed *PDE4D* (phosphodiesterase 4D), a cAMP‐specific phosphodiesterase reported to limit over‐syncytialization (Figure 2D) [34]. Finally, the mature STB‐c state was distinguished by tight junction inhibitors, including *ADAMTS6* (Figure 2D; Figure S2B). *ADAMTS6* disrupted cell–cell junctions and was previously identified as a distinct STB state marker [35, 36]. These findings suggest STB‐b nuclei balance syncytialization while STB‐c nuclei actively promote this process, maintaining dynamic equilibrium. Excessive syncytialization results in aged cytosolic material being released into maternal circulation, while restricted fusion leads to syncytial layer exhaustion [37]. Therefore, these three subpopulation dynamics may play a critical role in maintaining STB homeostasis. These STB states were validated by integrating with a recently published term placenta snRNA‐seq dataset [38] using scANVI [39] (Figure S2C). To confirm that our pseudo‐time assignment of the STB nuclei was valid, we transferred labels of published nuclei subtypes and aligned them on the previously established pseudo‐time axis. The distribution of the labels recapitulated the published distribution pattern (Figure S2D). STB state proportions showed no significant differences among the three groups (Figure 2E).

### 2.3 Maternal Obesity‐Associated Patterns That Are Shared or Different Between O‐A and O‐L Among STB Nuclei

**FIGURE 3:**
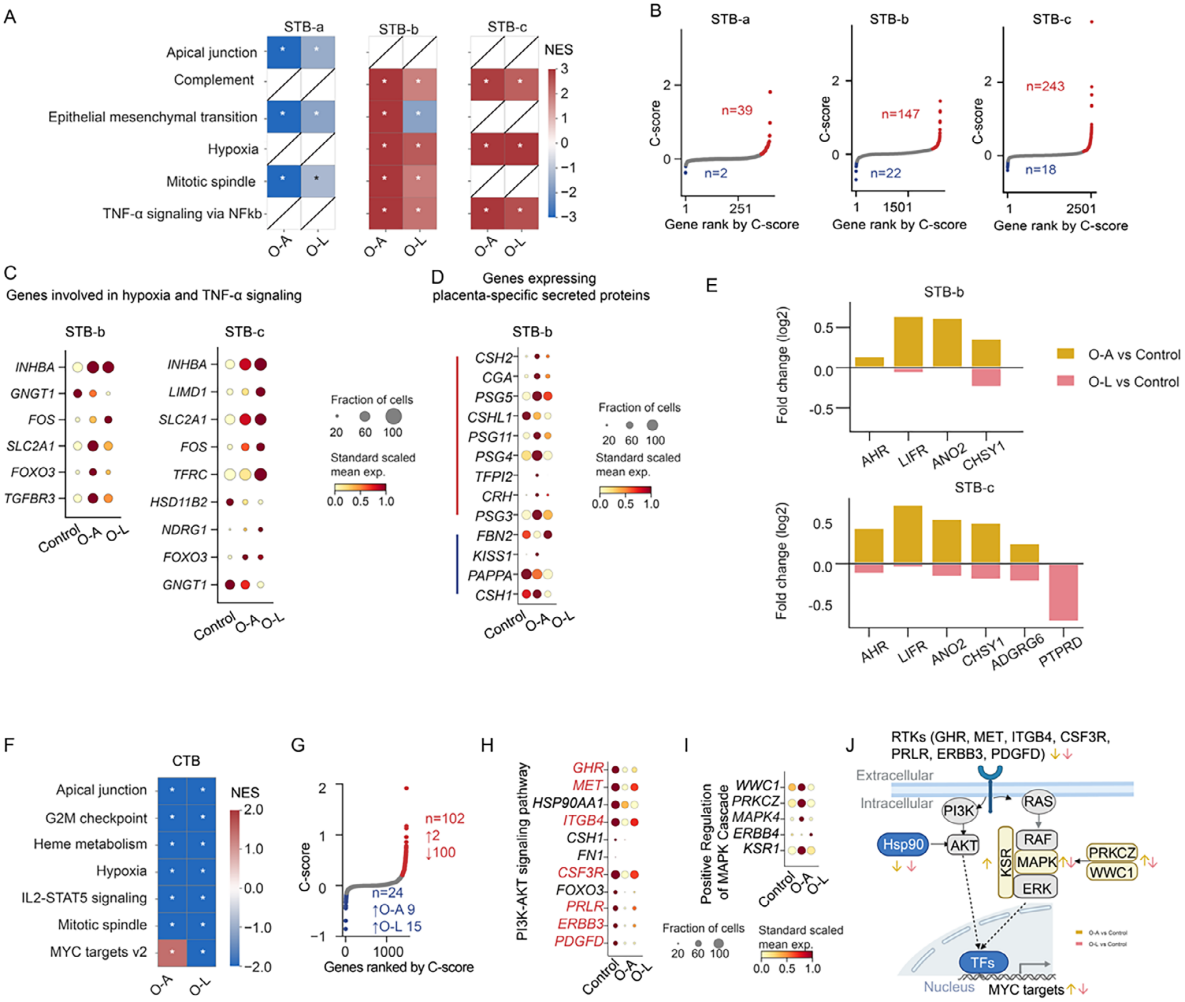
Gene expression changes in trophoblasts from mothers with obesity with or without fetal overgrowth. (A) Gene set enrichment analysis (GSEA) with the enrichment score of hallmark gene sets for O‐A versus Control and O‐L versus Control. NES: normalized enrichment score. *adjusted *p*‐value < 0.05. (B) Scatterplots of genes ordered by C‐scores in STB. (C) Dotplots showing the expression of the shared DEGs in O‐A and O‐L involved in the hypoxia pathway. (D) Dotplot showing the expression of the genes in STB‐b that encode placenta‐specific secreted proteins. (E) Barplots showing the fold changes of DEGs encoding for receptors, channels, or molecular sensors showing different maternal obesity‐associated patterns in O‐A and O‐L. (F) Gene set enrichment analysis (GSEA) with the enrichment score of hallmark gene sets for O‐A versus Control and O‐L versus Control. NES: normalized enrichment score. *indicates adjusted *p*‐value < 0.05. (G) Scatterplots of genes ordered by C‐scores in CTB. (H) Dotplots showing the expression of the genes involved in the PI3K‐Akt signaling pathway, and (I) positive regulation of the MAPK cascade. (J) Summary of genes involved in the pathways downstream of the receptor tyrosin kinase (RTK) signaling. The PI3K‐Akt signaling was reduced in CTBs from both obesity groups, while the MAPK signaling was upregulated in O‐A. Created with BioRender.com and modified with Adobe Illustrator.

The STB nuclei at different transcriptional states possibly vary in responses to surrounding signals. We first identified the underlying biological pathways in maternal obesity with or without fetal overgrowth utilizing hallmark gene sets from MSigDB [40]. Gene set enrichment analysis (GSEA) showed that O‐A and O‐L shared dysregulated pathways related to epithelial‐mesenchymal transition, apical junction, and mitotic spindle regions in STB‐a nuclei (Figure 3A, Table S3). STB‐b and STB‐c also showed several dysregulated pathways in both obesity groups, including complement, hypoxia, and TNF‐α signaling via NF‐kβ (Figure 3A, Table S3). GSEA with pseudo‐bulk data also showed these pathways being increased in STB‐b and STB‐c (Figure S2F). To determine genes that may contribute to these pathways, we intersected DEGs with the pathway gene sets, focusing on the hypoxia and TNF‐α signaling in STB‐b and STB‐c (Figure 3B). Among those shared genes, *FOXO3* and *FOS* have been reported as the crosstalk between hypoxia and TNF‐α signaling [41, 42, 43, 44]. Important transporters such as *SLC2A1* (i.e., GLUT1) and *TFRC* may mediate the interaction between hypoxia, nutrient transfer, and metabolism (Figure 3C) [45, 46]. *SLC2A1* is predominantly expressed in the STB to facilitate glucose uptake from the maternal circulation, and *TFRC* mediates cellular iron uptake. Expression of both is shown to be regulated by hypoxia [46].

Next, we used a previously published set of placenta‐specific secreted proteins from the Human Secretome Project [47]. A total of 12 genes showed shared maternal obesity‐associated patterns, among which most were upregulated except for *CSHL1* (Figure S2E). *CGA* encodes the alpha unit of human glycoprotein hormones, including hCG (human chorionic gonadotropin). Changes in hCG levels have been linked to adverse pregnancy outcomes, and their dysregulation highlights the detrimental effects of maternal obesity on the placenta [48]. A total of 5 genes encoding placenta‐specific secreted proteins showed different maternal obesity‐associated patterns between AGA and LGA. Specifically, *FBN2* was upregulated while *KISS1*, *PAPPA*, and *CSH1* were downregulated in O‐L (Figure 3D). Notably, *FBN2* encodes placensin, a glucogenic hormone highly expressed in human placentas that stimulates hepatic glucose secretion and cAMP production [49]. It was elevated in O‐L but decreased in O‐A, consistent with its increased expression in pregnancy complications such as gestational diabetes. *PAPPA* encodes a metalloprotease secreted by the human placenta that modulates IGF bioavailability. Its concentrations are inversely correlated with glycemia and the risk of developing gestational diabetes [50] in line with our observation of the greatest reduction observed in the O‐L group (Figure 3D). These differences in hormone regulation stem from the placental responses to maternal uterine conditions and may contribute to different fetal growth outcomes.

Furthermore, we investigated the expression of receptors or channels that sense the maternal metabolic signals. We found that a total of 7 genes were altered differently between O‐A and O‐L (Figure 3E). Among those expressed in both STB‐b and STB‐c, *AHR* encodes the aryl hydrocarbon receptor that senses the varied cellular environment, including endogenous tryptophan derivatives, an essential amino acid critical for placental and fetal development [51]. *LIFR* encodes the receptor of leukemia inhibitory factor, and LIFR deficiency leads to impaired placenta differentiation, reduced embryo viability, and increased birth weight in mice [52]. *ANO2* encodes a calcium‐gated chloride channel, and *CHSY1* is an enzyme for the synthesis of chondroitin sulfate in response to nutrient levels. These genes were only upregulated in O‐A but not in O‐L, suggesting an inadequate response in O‐L (Figure 3E).

### 2.4 PI3K‐AKT and MAPK Cascade Signaling Pathways Patterns in CTBs

Next, we analyzed the enrichment of hallmark gene sets in CTBs, the cell type that positions beneath STB. In contrast to STB, the hallmark gene sets were decreased in both O‐A and O‐L (Table S3). These included apical junction, mitotic spindle, G2M checkpoint, heme metabolism, hypoxia, and IL2‐STAT5 signaling (Figure 3F). However, Myc targets showed a divergent response with downregulation only in O‐L (Figure 3F). The MYC target pathway, critical for CTB proliferation, differentiation, and metabolic adaptation, is often suppressed by inflammation and hypoxia [53].

Next, we identified 102 genes showing shared maternal obesity‐associated patterns, among which 100 genes were downregulated (Figure 3G). Interestingly, the altered genes were significantly enriched in the PI3K‐Akt signaling pathway. Among them, *GHR*, *MET*, *ITGB4*, *CSF3R*, *PRLR*, *ERBB3*, and *PDGFD* were involved in receptor tyrosine kinase (RTK)‐mediated transduction of growth factor signals (Figure 3G). In addition, *HSP90AA1* is essential for stabilizing AKT activity, thereby facilitating the phosphorylation and inactivation of FOXO3 (Figure 3H) [54]. 24 genes showed different maternal obesity‐associated patterns between O‐A and O‐L (Figure 3G). Five of these dysregulated genes (i.e., *WWC1, PRKCZ, MAPK4, KSR1, ERBB4)* are involved in enhancing the MAPK cascade activation, a signaling pathway activated downstream of RTKs, similar to the PI3K‐AKT pathway (Figure 3I). *ERBB4* encodes an EGFR, which belongs to RTKs, and was reported previously to be expressed by CTB as a pro‐survival factor under hypoxic conditions [55]. Thus, our findings suggest that in O‐A, upregulation of the MAPK cascade might compensate for the downregulation of the PI3K‐Akt pathway, promoting downstream target expression, such as Myc targets (Figure 3F,J). However, this regulatory balance was likely absent in O‐L, which could explain the observed downregulation of Myc target genes in O‐L (Figure 3F). Altogether, the PI3K‐Akt and MAPK pathways, key downstream effectors of RTK signaling, are dysregulated in obesity and related to fetal overgrowth.

### 2.5 Maternal Obesity‐Associated Patterns in Non‐Trophoblast Cell Types: Shared or Different Between O‐A and O‐L

**FIGURE 4:**
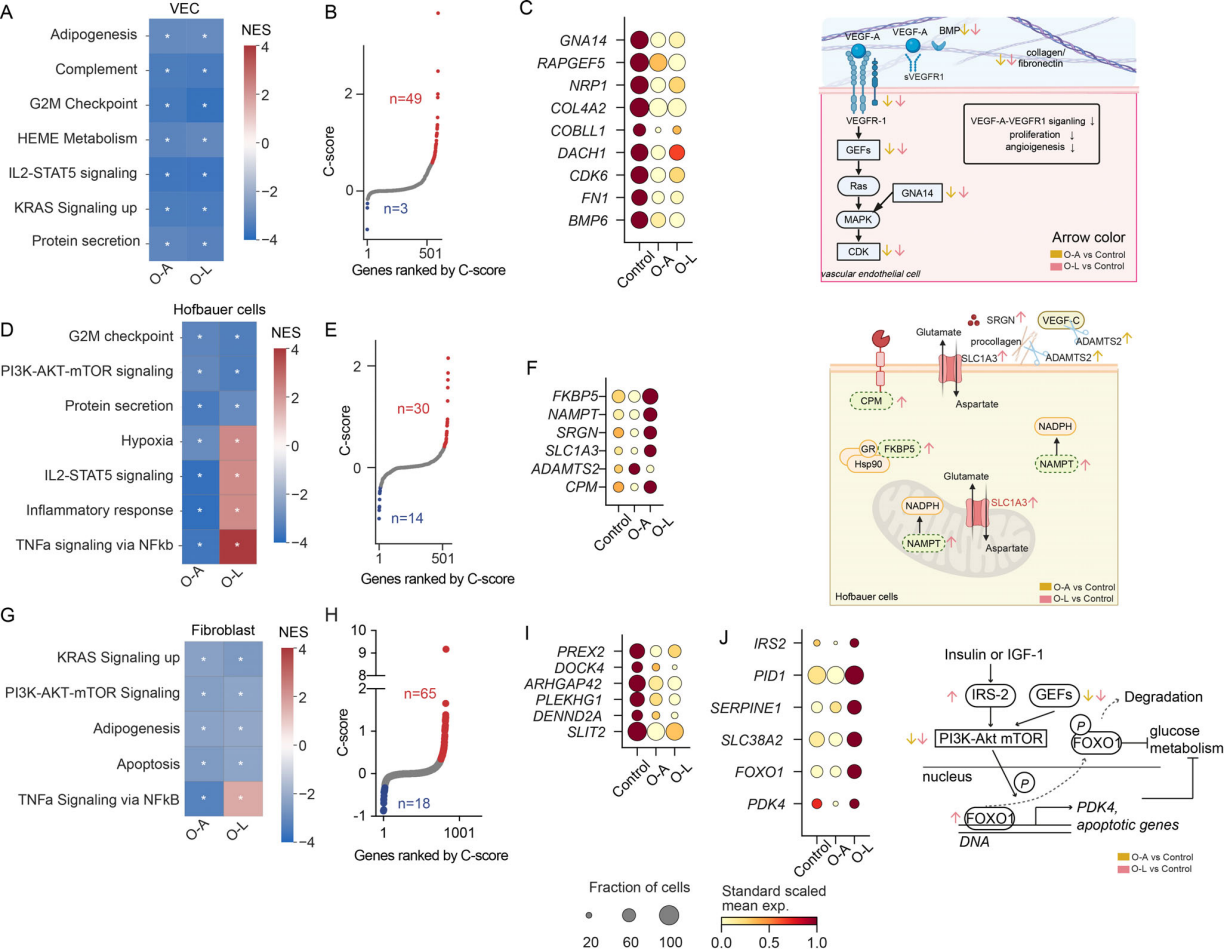
Gene expression changes in vascular endothelial cells, Hofbauer cells, and fibroblasts from placentas of mothers with obesity with or without fetal overgrowth. (A) Gene set enrichment analysis (GSEA) in vascular endothelial cells (VEC), with the enrichment score of hallmark gene sets for O‐A versus Control and O‐L versus Control. (B) Scatterplots of genes ordered by C‐scores in VEC. (C) Dotplot showing expression of genes relevant to FLT1 (VEGFR1) (left). Summary of genes involved in VEGFR1 signaling (right). (D) GSEA in Hofbauer cells, with the enrichment score of hallmark gene sets for O‐A versus Control and O‐L versus Control. (E) Scatterplots of genes ordered by C‐scores in Hofbauer cells. (F) Dotplot showing expression of genes involved in inflammatory response in Hofbauer cells (left). Summary of these genes in cellular context (right). (G) GSEA in fibroblast‐a, with the enrichment score of hallmark gene sets for O‐A versus Control and O‐L versus Control. (H) Scatterplots of genes ordered by C‐scores in fibroblast‐a. (I) Dotplot showing expression of genes related to GTPase activity that were changed in maternal obesity, and (J) the genes involved in PI3K‐Akt pathway and guanine nucleotide exchange factors that show different maternal obesity‐associated patterns between O‐A and O‐L. Summary of these genes within the PI3K‐Akt‐mTOR pathway (right). For (A), (D), (G), NES: normalized enrichment score. *adjusted *p*‐value < 0.05. For (C), (F), (I), the dot size is proportional to the fraction of cells that express the gene, and the color indicates the expression levels, standard‐scaled between 0 and 1. Illustrations created with BioRender.com and modified with Adobe Illustrator.

In VECs, gene sets related to complement, heme metabolism, IL2‐STAT5 signaling, and KRAS signaling were downregulated in both O‐A and O‐L (Figure 4A). These pathways are related to immune dysregulation, angiogenesis and metabolic stress [56]. Further examination of the genes within these pathways identified that 49 genes that had shared maternal obesity‐associated patterns, and 3 genes showed patterns that differ between O‐A and O‐L (Figure 4B). Nine genes were reported to be critical for regulating VEGFA‐VEGFR1 (i.e., FLT1) signaling in angiogenesis (Figure 4C). Among these, *GNA14, RAPGEF5, NRP1, COL4A2*, and *COBLL1* have been reported to be co‐expressed with *VEGFR1*, while *FN1* and *BMP6* have synergistic effects on VEGFA complex [57] (Figure 4C). The VEGFA complex and the adaptor encoded by NRP1 bind to VEGFR1 and promote angiogenesis [58] and inflammatory cell recruitment [59]. *FN1* dysregulation has been shown to affect VEGFA expression and relate to cellular apoptosis and autophagy in human umbilical vein endothelial cells [60, 61]. GSEA with pseudo‐bulk data confirmed the decreased angiogenesis pathway in O‐A and O‐L (Figure S2G).

In Hofbauer cells, the G2/M checkpoint signaling pathway, PI3K‐AKT‐mTOR signaling pathway, and protein secretion were suppressed in both obesity groups, suggesting dysregulation of cell proliferation, nutrient‐sensing, and tissue growth in maternal obesity (Figure 4D). These gene patterns might represent an adaptive mechanism in Hofbauer cells to curb excessive cytokines and nutrient stress in the placenta under obesity conditions. Notably, hypoxia, IL2‐STAT5 signaling, inflammatory response, and TNF‐α signaling via NF‐kβ were upregulated only in O‐L (Figure 4D). These gene expression patterns were indicative of inflammatory and metabolic stress that might impair placental homeostasis in O‐L. Next, we identified 30 genes regulated by maternal obesity (Figure 4E). Intriguingly, 14 genes showed different maternal obesity‐associated patterns between O‐A and O‐L (Figure 4F). Among the genes upregulated only in O‐L, *FKBP5* encodes for a member of the immunophilin protein family that stabilizes glucocorticoids receptor and promotes NFKβ‐mediated inflammation in response to reactive oxidative species [62]. *NAMPT*, also known as Visfatin, has a dual role as an enzyme and a cytokine regulating glucose homeostasis [63]. *SRGN* encodes a major secreted proteoglycan in macrophages and promotes TNF‐α secretion upon lipopolysaccharide stimulation [64]. Of note, *ADAMTS2* was only upregulated in O‐A but not in O‐L. *ADAMTS2* is a secreted metalloproteinase that processes procollagen into collagen, a critical step for extracellular matrix stability. Its expression has been implicated in the anti‐inflammatory response that helps maintain tissue homeostasis [65], which is consistent with increased inflammatory responses in O‐L.

Another essential cell type in the villous core is the fibroblast. It is critical for villi architecture, vascular development, immune regulation, and support of trophoblast functions [66]. In fibroblast, the KRAS signaling, PI3K‐AKT mTOR signaling, adipogenesis, and apoptosis gene sets were suppressed in both O‐A and O‐L, while TNF‐α signaling via NFKβ was upregulated only in O‐L (Figure 4G). We then identified 65 genes showing shared and 18 genes showing different maternal obesity‐associated patterns between O‐A and O‐L (Figure 4H). Six genes that encode guanine nucleotide exchange factors to regulate GTPase activity were decreased in both O‐A and O‐L (Figure 4I). These GTPase regulators control multiple small GTPases that are involved in the feedback loop in PI3K activation [67]. On the contrary, *IRS2*, *PID1*, *SERPINE1, FOXO1, and PDK4* were significantly upregulated in O‐L while downregulated in O‐A (Figure 4J). These genes are related to response to oxygen‐containing compounds (GO:1 901 700), including lipids (GO:0 033 993) and carbohydrates (GO:0 009 743). IRS2 is also a key mediator of cellular response to insulin stimulus (GO:0 032 869). FOXO1 can induce expression of *PDK4* [68], act as a key regulator for TNF‐α induced apoptosis, and inhibit glucose metabolism [69].

### 2.6 Cell–Cell Communication Patterns in Placental Villi That Are Shared or Different Between O‐A and O‐L

**FIGURE 5:**
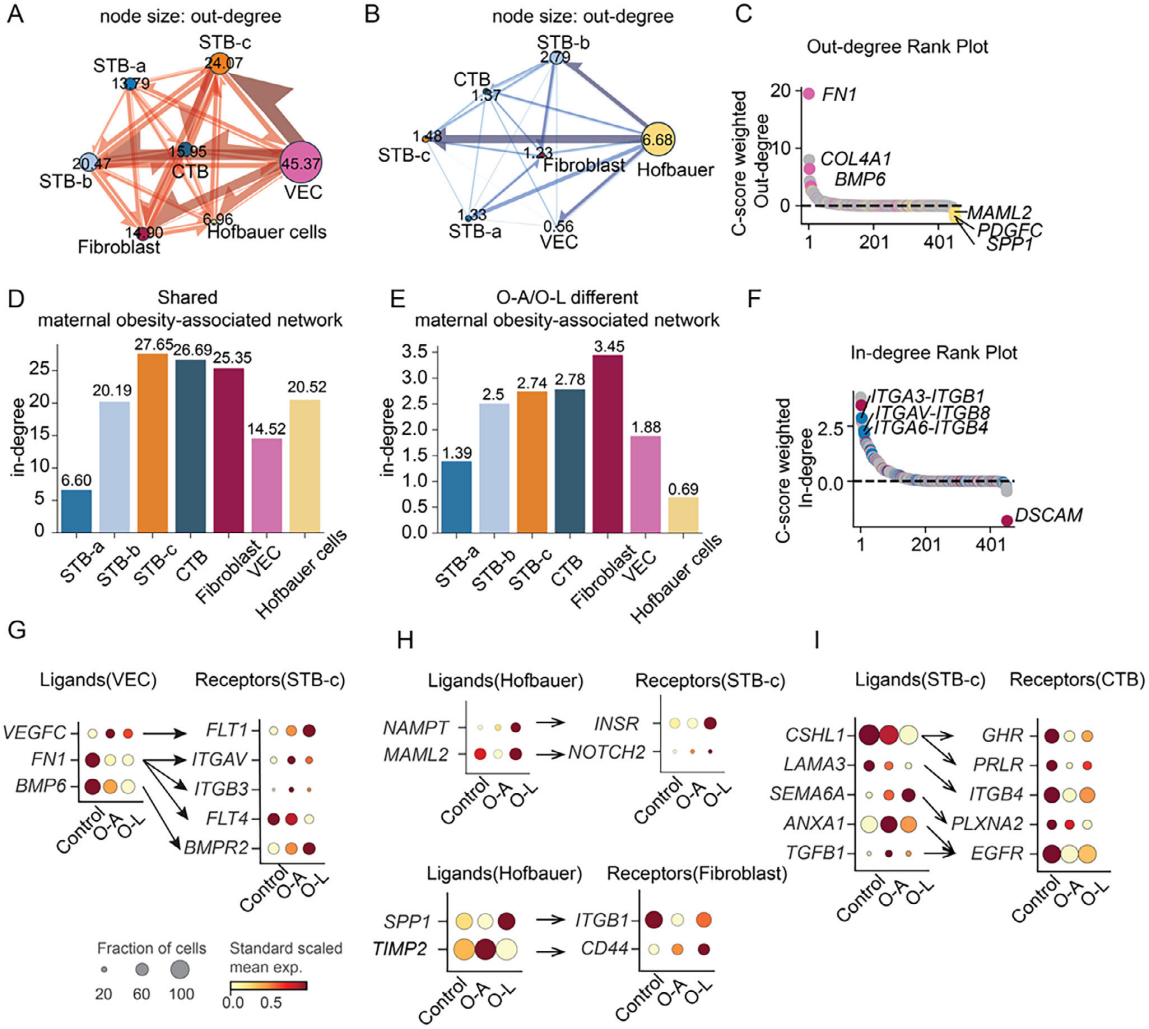
Dysregulated cell–cell communication networks of mothers with obesity, with or without fetal overgrowth. (A) Connectome ring plots for cell–cell communication built from differentially expressed ligand–receptor pairs showing shared maternal obesity‐associated patterns and (B) different between O‐A and O‐L. Different colors represent different cell types, node size indicating out‐degree, and edge direction goes from the sender cell type to the receiver cell type. (C) Out‐degree analysis indicates that the major or sender cells of ligand showing shared maternal obesity‐associated patterns and different between O‐A and O‐L. Pink dots represent the vascular endothelial cells, and the yellow dots represent the Hofbauer cells. (D) Barplot showing the in‐degree (the received signals) of each cell type from the shared maternal obesity‐associated network in (A). (E) Barplot showing the in‐degree (the received signals) of each cell type from the maternal obesity‐associated network that were different between O‐A and O‐L in (B). (F) In‐degree analysis indicates the major receiver cells with receptors. Red dots represent fibroblast‐a, and blue dots represent STB‐c. (G) Dot plot showing the expression of ligands from the VEC and receptors from STB‐c. (H) Dot plot showing the expression of ligands from Hofbauer cells and receptors from fibroblasts and STB‐c. (I) Dot plot showing the expression of the ligands from STB‐c and the receptors from CTB.

Our analyses have so far dissected the gene expression in each major placental cell type. To further explore intercellular communication and transcriptional coordination, we utilized the consensus resources from LIANA+ [70] to examine the ligand–receptor (L–R) interactions. We constructed a weighted directional network, mapping ligands from a cell type to receptors from another cell type (Figure S3). However, overall L–R interaction counts and strengths between intra‐villous cell types were similar across biological groups (Figure S3, Table S4). To assess L–R interaction regulation at a molecular level, we constructed a network where the strength of each L–R pair was quantified based on the shared or different expression patterns between O‐A and O‐L (using the C‐score calculation weighted by significance, Table S5). The interaction with positive regulation strength was shared maternal obesity‐associated network (Figure 5A). While L–R pairs with negative strength were altered differently between O‐A and O‐L, forming a maternal obesity‐associated network that differ between O‐A and O‐L (Figure 5B). In the shared network, VECs ranked highest in outgoing degree (45.37), followed by STB‐c (24.07) (Figure 5A). In contrast, in the network that differs between O‐A and O‐L, Hofbauer cells showed the strongest outgoing degree (6.68), with STB‐b (2.79) ranking next (Figure 5B). VEC‐derived ligands, including *FN1*, *COL4A1*, and *BMP6*, ranked highly as out‐degree genes in the shared maternal obesity‐associated network (Figure 5C). Hofbauer cells‐derived ligands, including *SPP1*, *PDGFC*, and *MAML2*, were prominent as out‐degree genes in the maternal obesity‐associated network that differed between O‐A and O‐L. Moreover, in‐degree scores across cell types represented the reception of signaling based on receptor expression (Figure 5D,E). We identified STB‐c ranked highest in the in‐degree (27.65), followed by CTBs (26.69), in the shared network (Figure 5D). In the network that differs between O‐A and O‐L, fibroblasts received the highest in‐degree interaction (3.45), with CTBs ranking second (2.78) (Figure 5E). Integrin complexes (*ITGA3‐ITGB1*, *ITGAV‐ITGB8*, *ITGA6‐ITGB4*) were highly ranked receptors expressed in STB‐c in the shared network, while *DSCAM* was the top‐ranked receptor expressed in fibroblast in the network that differ between O‐A and O‐L (Figure 5F).

The cell–cell communication patterns shared in O‐A and O‐L were mainly observed between VEC and STB‐c. The altered L–R pairs such as *VEGFC‐FLT1*, *FN1‐ITGAV*, *ITGB3, and FLT4*, and *BMP6‐BMPR2* (Figure 5G) play a role in vascular formation, tissue structure, and cell fate decisions. Moreover, top cell–cell communications patterns that differ between O‐A and O‐L were observed between Hofbauer and STB‐c cells. The altered L–R pairs included *NAMPT‐INSR* and *MAML2‐NOTCH2. NAMPT* functions as an intra‐NAD biosynthetic enzyme and extracellular inflammation mediator, and elevated extracellular NAMPT was reported in obesity [71]. Additionally, *SPP1‐ITGB1* and *TIMP2‐CD44* between Hofbauer and fibroblasts showed maternal obesity‐associated patterns that differed between O‐A and O‐L (Figure 5H). *SPP1* encodes a multifunctional glycoprotein, and the *Spp1* ^+^ macrophages emerged under obese conditions in mice [72]. *TIMP2* signaling was shown to stimulate fibroblast proliferation [73].

CTBs were also a prominent signaling receiver at vasculo‐syncytial membranes. Interestingly, we found that most L–R interactions occurred between STB‐c and CTB cells, involving growth‐promoting signaling pairs such as *CSHL1‐GHR/PRLR*, *LAMA3‐ITGB4*, *SEMA6A‐PLXNA2*, and *ANXA1/TGFB1‐EGFR* (Figure 5I). *CSHL1* encodes a human placental lactogen that can bind to the growth hormone receptor consisting of GHR and/or PRLR dimers. Additionally, epidermal growth factor (EGF) is required for proper control of growth and differentiation of CTB into STB [74] and altered EGFR expression has been associated with placental pathologies, including intrauterine growth restriction [75]. Receptors for these growth‐promoting ligands were decreased in CTBs, suggesting active adaptation to maternal obesity.

### 2.7 Partial Modeling the Obese Uterine Milieu by Co‐Culturing Adipose Spheroids and Trophoblast Organoids by a Customized Microfluidic System

**FIGURE 6:**
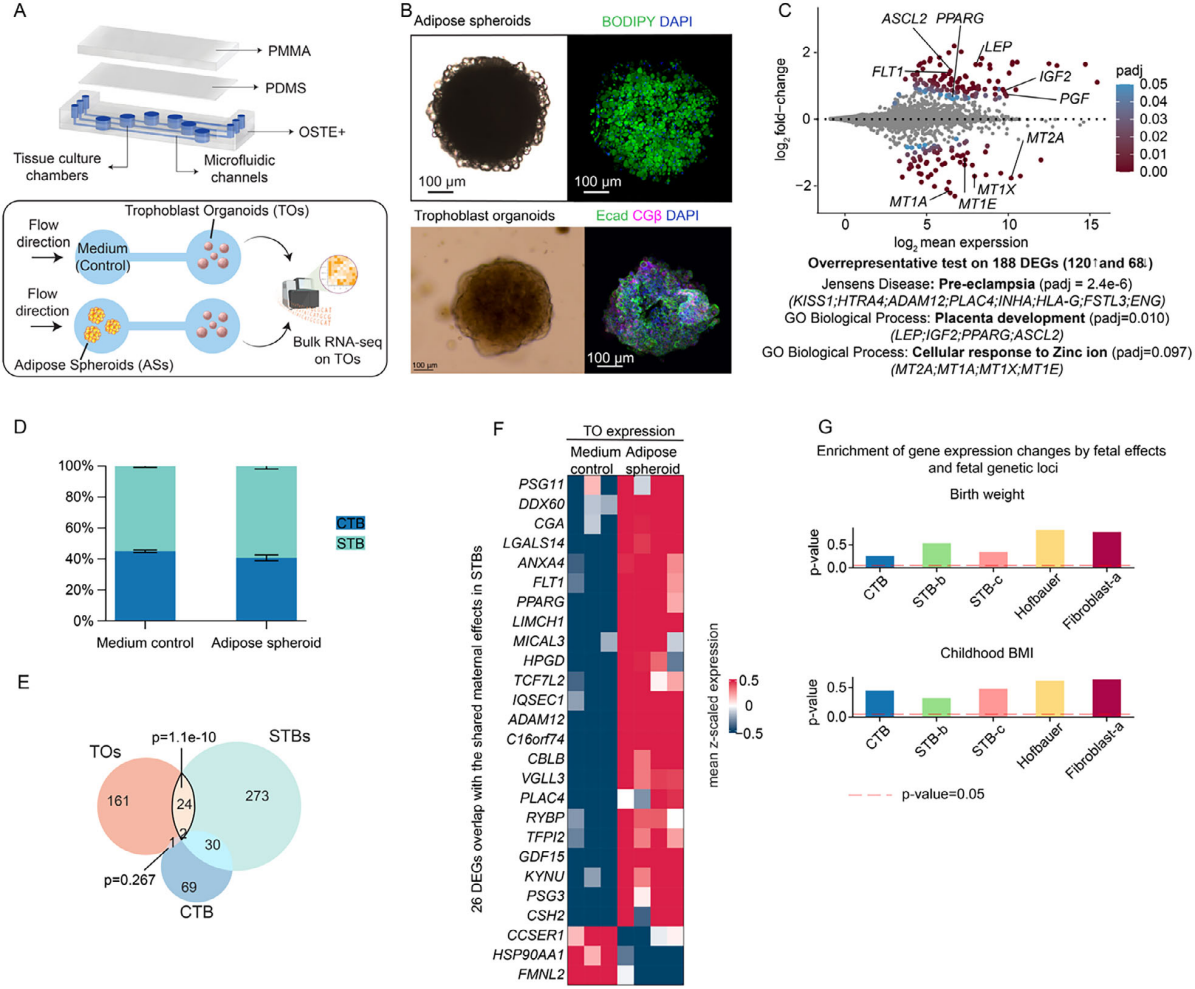
Trophoblast organoids co‐cultured with adipose spheroids partially reflected the transcriptional changes observed in STB of mothers with obesity. (A) Design of the microfluid device and co‐culture experiment of Trophoblast Organoids (TOs) and adipose spheroids (ASs). (B) Representative images of ASs (up; bright‐field image and confocal image stained with BODIPY and DAPI to highlight neutral lipids and nuclei, respectively) and TOs (bottom; bright‐field image and confocal image stained with E‐cadherin, CGβ, and DAPI to highlight CTBs, STB, and nuclei, respectively). Images were captured with 10× magnification. (C) MA plot of gene expression in TOs co‐cultured with ASs (*n* = 4) compared with TOs cultured without ASs (*n* = 3). (D) Stacked bar plot showing the proportion of different cell types based on the single‐nucleus RNA‐sequencing reference [115]. The proportion reveals no significant cellular (nuclear) composition in trophoblast organoids cultured with medium control or adipose spheroid. (E) Venn diagram showing the overlap of the differentially expressed genes in the microfluidic co‐culture experiment and trophoblast in maternal obesity groups. (F) Expression heatmap for the 26 overlapping genes in (E). (G) Enrichment between cell–type–specific differentially expressed genes showing different maternal obesity‐associated patterns between O‐A and O‐L and genomic variants implicated by GWAS for birth weight [81] and childhood BMI [82] computed with MAGMA [85].

Although it is well‐established that maternal obesity affects placental function and offspring outcomes, the role of different maternal organs and tissue–tissue communication with the placenta remains unclear. Therefore, we investigated whether any observed expression patterns in the trophoblasts could be attributed to communication between the adipose tissue and the trophoblasts. We adopted a microfluidic co‐culture system with medium flowing from primary adipose spheroids (ASs) to human term placenta‐derived trophoblast organoids (TOs). The main component of the fluidic device contained interconnected tissue chambers, arranged in pairs, enabling parallel perfusion for triplicate experiments [76]. To ensure gas permeability and conformal contact, a polydimethylsiloxane layer—never in direct contact with the culture media—was applied over the fluidic layer and an additional layer of poly(methyl methacrylate) was mounted on the top. It provided a stable chip‐to‐world connection and ensured sealed perfusion when inlet and outlet connectors are assembled (Figure 6A). The medium was optimized for glucose, insulin, and free fatty acids in consistent with its prior use for ASs culture [77]. The first chamber pair of the microfluidic chip was designated for a medium control condition without ASs, and in the second chamber pair, we placed ASs [77] before seeding them into the device for a 4.5‐day co‐culture. The ASs were established from white adipose tissue of female origin and cultured for a total of 17 days before being introduced into the microfluidic device. ASs displayed large lipid droplets stained by BODIPY, consistent with previous findings (Figure 6B) [77]. TOs exhibited a typical morphology with CTBs forming the outer layer with E‐cadherin staining, and STB embedded inside the inner layers, with CGβ staining, as shown previously in Matrigel‐embedded cultures (Figure 6B) [78, 79].

To examine how TOs were affected by secreted factors from ASs, we performed bulk RNAseq of the TOs. After the continuous exposure to obese ASs, the TO transcriptome was altered, with 188 differentially expressed genes (DEGs; 120 upregulated and 68 downregulated) when compared to the medium control condition. While bulk RNA‐seq may miss genes altered in opposite directions in CTB and STB, the detected DEGs (fold change ≥ 2, false discovery rate ≤ 0.05) have a low false‐discovery rate. Among the upregulated genes, *LEP*, *IGF2*, *PPARG*, and *ASCL2* are related to placenta development (GO:0 001 890), while *KISS1*, *HTRA4*, *ADAM12*, *PLAC4*, *INHA*, *HLA‐G*, *FSTL3*, *ENG* are related to pre‐eclampsia (from Jensen's Diseases Database [80]) (Figure 6C). On the contrary, metallothionein‐encoding genes *MT1A, MT1E, MT1X*, and *MT2A* were significantly downregulated, suggesting impaired cellular response to ions such as zinc (GO:0 071 294) (Figure 6C).

Transcriptome‐based cell type deconvolution [81] confirmed that TOs maintained a consistent composition of STB and CTB across conditions. In the medium control, the median STB proportion was 55.3% (range 53.4%–56.3%); in the ASs group, the median was 59.2% (range 55%–63.6%), with the remainder being CTB (*p* = 0.2286 by Mann–Whitney *U*‐test, Figure 6D). Next, we compared 188 DEGs found in TOs with DEGs of trophoblast cell types from our snRNA‐seq analysis (Table S6). A total of 27 overlapping genes were identified, with 26 shared with STB (Figure 6E). Interestingly, these genes showed shared maternal obesity‐associated patterns. The limited number of overlapping DEGs might be explained by the missing factors like chronic inflammation and hypoxia among the full complexity of maternal obesity. Among these genes, many were highly expressed in TOs co‐cultured with AS (Figure 6E). These included genes encoding placenta‐specific secreted proteins [47], including *CGA*, *PSG3, PSG11*, *CSH2*, and *TFPI2* (Figure 6F). *LGALS14*, important for membrane integrity, was also upregulated (Figure 6F). The patterns that differ between O‐A and O‐L were not recapitulated by adipose tissue‐derived signaling.

We investigated whether the identified patterns that differ between O‐A and O‐L were linked to genetic loci. We examined previously identified by genome‐wide association studies (GWAS), associated with birth weight (fetal GWAS) [82], childhood BMI [83], and placenta‐specific quantitative trait loci (QTL) linked with birth weight [84, 85]. We used MAGMA, to analyze the statistical association between the aggregate genetic risk at the gene level [86]. To ensure reliable results, we excluded cell types from this analysis with fewer than 10 DEGs. For the remaining placental cell types, we did not find strong associations between genes that differed between O‐A and O‐L and risk genes at the genetic variation level [82, 83, 84, 85] (Figure 6G).

## 3 Discussion

**FIGURE 7:**
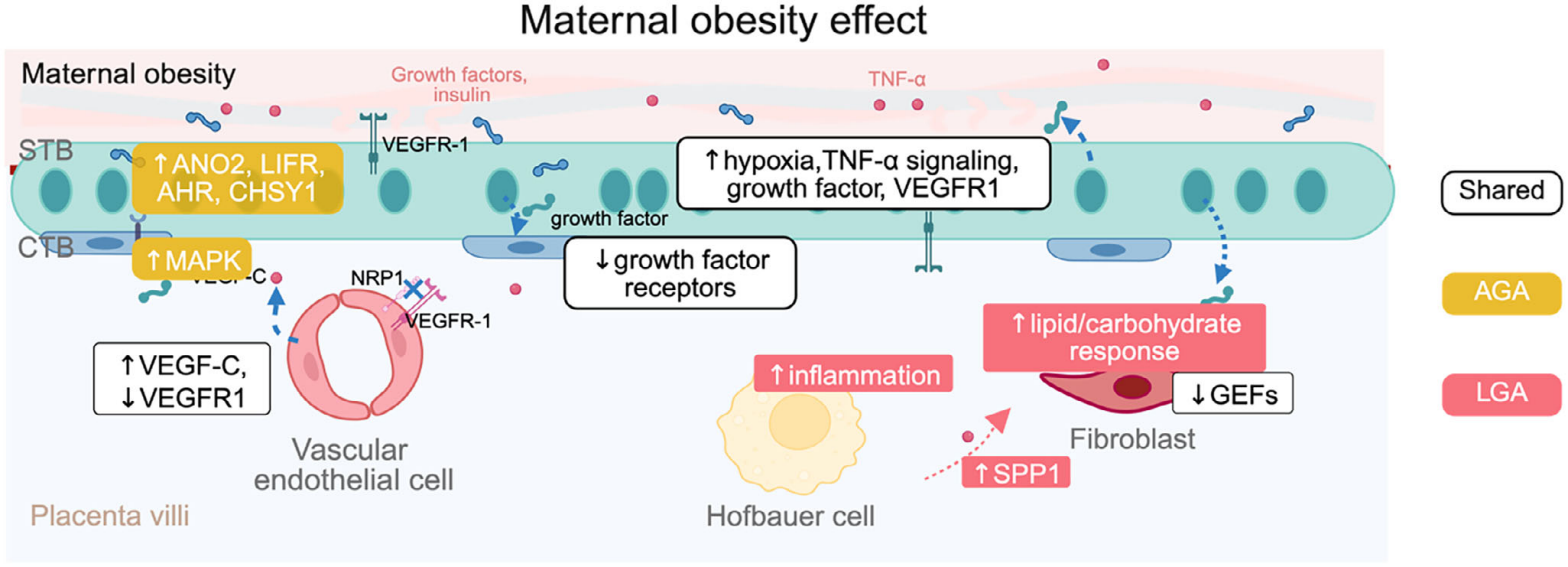
Schematic summary showing hypothesized maternal obesity‐associated patterns that were shared or different between O‐A and O‐L, based on transcriptomic inference. Created in BioRender.

Maternal obesity during pregnancy is associated with adverse outcomes for both the mother and offspring. A deeper understanding of the molecular mechanisms driving placental responses is crucial for future interventions and therapeutic development. In this study, we examined placentas of women with obesity who did not develop other pregnancy complications but delivered either AGA or LGA infants. This could help minimize confounding factors and allow for a clearer understanding of inherent placental alterations. Given that the human placenta is a highly specialized organ with distinct cell types, including the multinucleated STB layer, we utilized snRNA‐seq to reveal cell‐type‐specific transcriptional responses to maternal obesity and fetal growth. Notably, we were able to identify a set of shared and different maternal obesity‐associated patterns between O‐A and O‐L. We inferred cell–cell communication that provides hypotheses about key cell types and pathways underlying these two patterns. Notably, VECs, STB, Hofbauer cells, and fibroblasts played prominent roles in such communications. These hypotheses are summarized in Figure 7.

Previous studies have shown that altered metabolites, hormones, and cytokines—such as IL‐6 and TNF‐α—can be sensed by trophoblast cells in cases of maternal obesity, thereby influencing trophoblast proliferation, nutrient uptake, and transport [87, 88]. Furthermore, activation of phospholipase A2 by TNF‐α and leptin in human trophoblast cells has been proposed as a mechanism contributing to excessive fetal fat accumulation and neonatal adiposity [89]. A recent study examined 14 PROGENy pathways and reported hypoxia upregulation in fetal‐side STB non‐proliferative CTBs, one cluster of fibroblast, VEC, one cluster of fetal macrophages, and maternal side EVT [28]. In line with this, and extending the analysis with the broader MSigDB Hallmark gene sets [40], our findings reveal that the STB exhibits an enrichment of pathways related to elevated hypoxia and TNF‐α signaling via NF‐kβ as shared maternal obesity‐associated patterns. These patterns were potentially driven by alterations in genes involved in the cross‐regulations of these pathways, such as *FOS*, *FOXO1*, and *SLC2A1*. *SLC2A1* is of particular interest as it encodes GLUT1 to promote glucose uptake. Similar to other metabolic organs, there could be an interplay between hypoxia, inflammation, and excessive nutrient transport in the STB. Interestingly, we have recently demonstrated how mild hyperglycemia can still induce placental hypoxia in mice, suggesting excessive hypoxia as a common placental signature in maternal metabolic disorders [90].

Notably, as a shared maternal obesity‐associated pattern, CTBs exhibited downregulated genes involved in PI3K‐Akt signaling pathways, particularly those RTKs associated with the *GHR*, *PRLR*, and *CSF3R*. The altered expression of these receptors in CTBs is likely an active adaptation to the altered ligands expressed in the STB. When analyzing these L–R interactions between two cell types within placental villi, we found that mature STB‐c altered the expression of growth‐promoting ligands while the growth factor receptors in CTBs [91, 92] were downregulated. Likely, downregulation of these receptors in CTBs, followed by PI3K‐Akt signaling pathways, could limit the tissue growth signaling in maternal obesity.

Importantly, we further uncovered the different maternal obesity‐associated patterns between O‐A and O‐L. Notably, TNF‐α signaling and inflammatory response were decreased in the Hofbauer cells in O‐A, whereas they were prominently increased in O‐L, implying that LGA outcome in maternal obesity is associated with inflammation. Specifically, *NAMPT* and *SC1A3*, which play a dual role in this inflammatory response and metabolism [93] were exclusively upregulated in O‐L. These genes likely facilitate the immune‐metabolic modulation by Hofbauer cells. Moreover, L–R network analysis revealed that Hofbauer cells expressed ligands in a birth weight‐dependent network. The Hofbauer cells in O‐L highly expressed the ligand *SPP1*. Notably, SPP1^+^ macrophages have been reported to localize in hypoxic areas in tumors, to support tumor progression by promoting cell survival, angiogenesis, and immunosuppression [94]. In addition, dysregulated Hofbauer polarization and immune regulation were implied in regulating feto‐placental angiogenesis [95, 96, 97]. Therefore, responses of Hofbauer cells to a hypoxic and inflammatory uterine environment in maternal obesity may relate to LGA outcome.

An increase in TNF‐α signaling in fibroblasts was also observed in O‐L. This signaling may impair insulin action on fibroblasts by inhibiting the activation of IGF‐1/insulin hybrid receptors [98]. In fibroblasts, the increase of TNF‐α signaling and genes involved in insulin receptor signaling, such as *IRS2* may be related to LGA outcome in maternal obesity. In line with this, we identified fibroblasts ranked highest in the in‐going degree of the maternal obesity‐associated network that differed between O‐A and O‐L. The most expressed ligand correlated with it was macrophage‐derived *SPP1*, which has been shown to activate myofibroblasts in fibrosis [99]. Fibroblasts and other stromal cells in the term placenta have been understudied in prior research; our analysis highlights the transcriptional responses of these cells to maternal obesity, particularly with fetal overgrowth. Interestingly, TNF‐α signaling was increased in STB of both obesity groups, but only increased in Hofbauer cells and fibroblasts in O‐L. This may indicate that the STB response to TNF‐α is predominantly shaped by direct exposure to maternal circulating inflammatory signals, whilst within the placental villi, these maternal signals need to be dependent on fetal growth.

When investigating the intercellular communication network, we found that the STB ranked the highest in in‐degree in the shared maternal obesity‐associated network. Whether these signals originate from maternal circulation or villous cells cannot be concluded from the snRNA‐seq data. In addition, we used a novel microfluidic system to co‐culture TOs and ASs. This confirmed that certain signals were likely from adipocytes. While not a comprehensive model of maternal obesity, our microfluidic co‐culture system displayed several advantages for studying the functional impact of tissue communication. The unidirectional media flow from ASs to TOs simulated adipose‐related effects on the trophoblasts. As DEGs in TOs exhibited a greater overlap of altered genes in the STB than CTB to maternal obesity, including *FLT1* and *PPARG*, this further suggests the key role of STB regulation by the maternal environment. The unidirectional flow design of the co‐culture system does not account for signaling from fetal tissues, which limits the interpretation of the differences in O‐A and O‐L observed in vivo. Notably, these birth weight‐dependent patterns are not among those genetic loci identified by GWAS of birth weight and childhood obesity. The lack of overlap with GWAS‐identified loci suggests that the fetal growth‐associated transcriptional patterns observed in our study are likely driven by non‐genetic factors, such as environmental or metabolic cues, rather than heritable genetic variants. Nevertheless, to fully evaluate genetic contributions to placental responses, more comprehensive approaches like large‐scale eQTL studies for birth weight and functional validations with embryo models are needed.

Although this study provides new insights into maternal obesity‐associated expression patterns in the placenta and among them, the patterns that are birth weight‐dependent, it has several limitations. The pseudo‐bulk approach would likely miss some cell‐type‐specific expression changes, especially in placenta snRNA‐seq data where only STB, CTB, and VEC were >5% of nuclei and other cell types were rare. We did not perform protein‐level validation or functional assays of the promising molecular targets or intercellular communication findings. Moreover, even if our samples are only from placental villi, without decidua or chorion membrane, our approach did not preserve spatial context within the villi. And the inferred cell–cell communication networks rely on the assumption that involved cell types are within effective signaling proximity. While prior histological studies have shown that Hofbauer cells reside near feto‐placental vessels and basal membrane of the trophoblast layer [96], and vasculosyncytial membrane where endothelial cells and STB are in proximity [100], direct evidence of the predicted signaling interactions remains to be established. Integrating spatial transcriptomics or imaging, proteomic, and functional approaches is warranted in the future to experimentally validate the proposed hypothesis including the communications between STBs, fibroblasts, and Hofbauer cells. Specific functional studies could include SPP1 and TNF pathway modulation to primary placental fibroblasts, inflammatory cytokine secretion assays, and extracellular matrix remodeling assays to directly test the role of these signaling pathways in mediating maternal obesity‐induced placental dysfunction. Moreover, immunohistochemical staining for VEGFR and VEGFA expression, placental vascular morphometry analysis, and Doppler ultrasound assessment of uteroplacental blood flow could be performed to validate endothelial cell dysfunction and its functional consequences. Of note, while our microfluidic co‐culturing system modeled one facet of the obesity environment with adipocytes, the obesity environment is complex with other facets, including chronic low‐grade inflammation and hypoxia, as suggested by our transcriptomic data and prior studies. Therefore, the results from the microfluidic co‐culture provide supportive validation of subsets of our in vivo observations. To systematically model this complex environment, future studies could incorporate other factors, like hypoxic stress, for example, by using dimethyloxalylglycine to stabilize HIF1A. Extending on our current setup with only one obesity condition, establishing versatile conditions is needed to study the heterogeneity of obesity.

In conclusion, our study demonstrates intricate cell type‐specific expression patterns within the placenta, driven by critical pathways such as hypoxia, nutrient transport, inflammation, and TNF‐α signaling underlying maternal obesity and birth weight outcomes. Given the rising prevalence of global obesity, these mechanistic insights will deepen our understanding of placental response and birth weight outcomes.

## 4 Materials and Methods

### 4.1 Experimental Design

The Institutional Ethics Committee for Research on Humans of the Faculty of Medicine, University of Chile (Protocol No. 033‐2013, 201‐2017, 236–2020) reviewed and approved the protocol. At the time of recruitment, each participant received all information regarding the study, voluntarily enrolled, and signed the informed consent. Women were recruited before delivery at the Maternity Service of the Hospital San Juan Dios, West Health Division, Santiago, Chile, between 2014 and 2022. From the 328 placentas collected during this period, women with pregnancy complications such as hypertension, preeclampsia, thyroid disorders, GDM, preterm delivery, a fetus with malformations or chromosomal aberrations, and those with pregestational antecedents of diabetes, polycystic ovary syndrome, infertility, and assisted fertilization (IVF or ICSI) as well as those who declared to smoke, drink alcohol or take drugs during the pregnancy were excluded from this study. Women with normal pregestational weight (BMI between 18.50–24.99 kg/m^2^) or pregestational obesity grade I (BMI between 30–35.7 kg/m^2^) aged between 18 and 34 years old, who delivered by vaginal delivery were further selected. Birth weight appropriate to gestational age (AGA) was defined as a birth weight more than the 10th and 90th percentile, and birth weight large to gestational age (LGA) was defined as a birth weight above the 90th percentile of the sex‐specific and gestational age‐specific reference mean [6]. For single‐nucleus analysis, placenta samples from control (normal‐weight mothers and AGA babies, *n *= 4), O‐A (mothers with obesity and AGA babies, *n *= 4), and O‐L (mothers with obesity and LGA babies, *n *= 4) were selected. The maternal age, height, and gestational age were not different between groups. Offspring sex was considered a factor when comparing expression between groups.

### 4.2 Sample Collection

After delivery, placentas were collected as previously described [29] and snap frozen immediately in liquid nitrogen. Briefly, placentas from full‐term pregnancies were collected immediately after delivery and processed within 30 min. Each placenta was sectioned transversely using a sterile scalpel near the cord insertion site (approximately 5 cm), and the villous part was used for snRNA‐seq. The samples were then conserved in −80°C freezers until transport to Sweden, when they were kept on dry ice and then stored again in −80°C freezers. Newborn data such as sex, weight, height, and head circumference were recorded. Placental efficiency was calculated as the ratio of neonatal weight (*g*) to placental weight (*g*).

### 4.3 Nucleus Isolation

The protocol for nucleus isolation was adapted from the protocol optimized for human placenta samples by Ludivine Doridot and her team (Institut Cochin, France). Briefly, 30 mg of frozen placenta was chopped into small pieces on dry ice and added to a douncer with cold NP‐40 lysis buffer containing RNase inhibitor. After 10 min of incubation on ice, the lysis buffer was diluted in wash medium (cold PBS with 2% BSA and RNase inhibitor), and 10 strokes with the loose pestle were performed. Samples were further filtered (100 µm) and centrifuged at 600 g for 5 min at 4°C. The Pellet was resuspended in cold wash medium and then further filtered (40 µm) and centrifuged again. After resuspension in nuclei resuspension buffer (1% BSA and RNase inhibitor solution in DPBS), the pellet was filtered one last time (20 µm).

The number of isolated nuclei was determined using trypan blue staining and an automated cell counter (Biorad, TC20), and the quality of nuclei was assessed by visualization under a microscope (EVOS XL Core, ThermoFisher Scientific).

### 4.4 10× Genomics Library Preparation and Sequencing

Suspension of good‐quality nuclei was processed according to the manufacturer's protocol for the Chromium Single Cell 3’ kit v3.1 (10× Genomics). Library preparation was performed to obtain between 1000 and 10 000 nuclei per reaction. Libraries were then sequenced to obtain 90 GB of data (paired end, 150 bp) per reaction on the Illumina NovaSeq 6000 at Novogene, UK.

### 4.5 snRNA‐Seq Processing

Gene counts were obtained by aligning reads to the GRCh38 genome using Cell Ranger software (10x Genomics). We used scanpy [101] to process and cluster the expression profiles and infer cell identities of major cell classes. Cellbender [102] was used to remove background noise. In addition, using Scrublet [103], cells that were labeled as doublets were removed.

From raw UMI counts, the analytical Pearson residue [104] was applied, and highly variable genes shared across batches were selected as features. Principal component analysis (PCA) was performed, and the first 30 components were used to construct the nearest‐neighbor distance matrix. Nuclei were clustered using the Leiden algorithm applied to this matrix. We annotated cell types using previously published marker genes single‐cell RNA‐sequencing data [105].

For the Pseudo‐time analysis of trophoblast nuclei, Slingshot [106] was applied. Slingshot was designed to identify a minimum spanning tree. In this setting, pCTB was assigned as an initial cell state, and STB was classified as a terminal cell state.

We integrated the syncytiotrophoblast data generated in this study with the syncytiotrophoblast data from the public human healthy term placenta snRNA‐seq data [38] into the same embedding using scANVI [39]. We transferred their label onto our nuclei and compared the transferred labels with our labels using the silhouette metric. The silhouette metric's values range from [−1, 1], with 1 indicating distinct labels, 0 indicating overlapping labels. Thus, the absolute value of the silhouette width was used to measure how well labels were mixed.

We applied a Bayesian model in scCODA [107] for testing the compositional differences across groups. pCTB population was selected as the reference cell type, which is assumed to be unchanged in absolute abundance, and parameter inference was calculated via Hamiltonian Monte Carlo sampling.

### 4.6 Differential Expression of Obesity Groups Versus Control

We performed differential expression analyses using a Wald test with a negative binomial regression model using the formula “∼1 + group + sex + log_number_of_genes” on raw counts. Such analysis was deployed in the R Seurat package [107]. By default, in the FindMarkers function in Seurat, genes with more than one count in at least 1% of cells were considered. This rendered 6000 to 7000 genes to be analyzed. FDR was estimated with the Benjamini–Hochberg method. The statistics from this analysis were used as the input in the following method section.

For the gene set enrichment analysis, we ranked the genes in O‐A versus Control and O‐L versus Control differential gene expression analysis, respectively, by fold changes. We then performed GSEA [108] to get the normalized enrichment scores. Specifically, an enrichment score was calculated by walking down the list of genes, increasing a running‐sum statistic when a feature in each hallmark gene set was encountered and decreasing it when it was not. The final score was the maximum deviation from zero encountered in the random walk. Finally, a normalized score was obtained by computing the z‐score of the estimate compared to a null distribution obtained from N random permutations. The *p*‐values were adjusted by the BH method [109] to control the false discovery rate.

Gene Ontology enrichment analyses were performed using Enrichr. Specifically, Fisher's exact test was used to test the overrepresentation of the gene sets of interest from this study in known gene sets from the MsigDB Hallmark database. The *p*‐value from the test was adjusted with the BH method [109].

For sample‐level GSEA analyses, we aggregated counts across cells of the same type within each sample to create pseudobulk data and applied the implementation of DESeq2 and fast GSEA using decoupler [110].

### 4.7 Scoring the Shared or Different DEGs Between Comparisons

To compare gene expression patterns between the two obesity groups (O‐A and O‐L) and controls, we computed a continuous *“C‐score”* for each gene. This score combined the log_2_ fold‐change and the FDR from each comparison to quantify how similarly or differently a gene was regulated in O‐A versus O‐L. Importantly, our primary analysis considered whether each gene's expression change was in the same or opposite direction in two comparisons (i.e., shared vs. different regulation). The C‐score served as a supplementary quantitative measure of this pattern. Positive C‐scores denoted that a gene was either upregulated in both O‐A and O‐L or downregulated in both (shared maternal obesity‐associated patterns), whereas negative C‐scores indicated that a gene's expression was increased in one group but decreased in the other (different maternal obesity‐associated patterns between O‐A and O‐L). The larger the absolute C‐score, the greater the fold changes and adjusted *p‐*value in the shared or different gene expression patterns. This approach complemented traditional overlap analysis by providing a continuous difference between comparisons, rather than a binary classification based on arbitrary thresholds. Formally, we define:

C−score=Magnitude×Ratio

$$Magnitude=\left|E1\times \;wFDR_{E1}\left|+\right|E2\times \;wFDR_{E2}\;\right|$$

$$Ratio=\left\{\begin{array}{c} \frac{\max\left(\left|E1\left|,\;\right|E2\right|\right)}{\left|E1-\;E2\right|+1},\;E1\times \;E2>0\\ -\frac{\left|E1-\;E2\right|}{\max\left(\left|E1\left|,\;\right|E2\right|\right)+1},\;E1\times \;E2\leq 0 \end{array}\right.$$

$$wFDR=\left\{\begin{array}{c} 1,\;FDR<0.05\\ \frac{log_{10}FDR}{log_{10}0.05},\;FDR\geq 0.05 \end{array}\right.$$

$$\begin{array}{ccc} E1 & ≔\; & log_{2}FC\;or\;normalized\;enrichment\;score\;for\;O\\ & & -A\;\mathrm{versus}\;Control,\\ \;E2 & ≔\; & log_{2}FC\;or\;normalized\;enrichment\;score\;for\;O\\ & & -L\;\mathrm{versus}\;Control \end{array}$$

A positive score indicated that the genes were both increased and decreased in the two comparisons. The higher the score, the more similar and larger the fold changes were. Conversely, a negative score indicates that the gene was oppositely expressed in the two obese groups compared to the normal weight group. This means that it was increased in one comparison group and decreased in the other. The more negative the score, the more divergent and larger fold changes the gene had. In this algorithm, genes with zero‐fold changes were scored as zero.

To measure the significance of the scores of genes that were differentially expressed (score ≠ 0), we performed a permutation test. For each cell type, the fold changes and FDRs in each comparison were permuted 40 000 times if the number of genes was over 200; otherwise, they were permuted (number of genes)^2^ times. The *p*‐values were calculated for the observed scores as the frequencies of the permuted scores > the observed score when the observed score was positive or as frequencies of permuted scores < the observed score when the observed score was negative. A one‐sided *p‐*value < 0.05 was used for significance.

Using public RNA‐seq datasets, we demonstrated its utility to support the fold‐change direction framework to identify shared or different patterns:

1. Hindlimb unloading/reloading mouse model [111], and thus should have low negative C‐scores, indicating different expression regulations in the unloading and reloading process. The genes that were experimentally validated with such a different regulation pattern were among the 3 genes that had the lowest C‐score.
2. KO/knockdown dataset snRNA‐seq [112]. From the pooled CRISPRi and knockout perturbation dataset, the C‐score between the contrasts KO versus control and CRISPRi versus control detected shared transcriptional responses, which were reported in the original publication.

Detailed scripts, explanations, and benchmarking results are publicly available in our GitHub repository (denglab‐ki.github.io/cscore/).

### 4.8 Cell–Cell Communication (CCC) Network Analysis

Ligand–receptor interactions across cell type pairs were inferred using the consensus ligand–receptor database from LIANA+ [70]. The ligand–receptor edges were weighted by the sum of C‐scores for the corresponding genes obtained with the method stated in the preceding section. This generated the cellA–cellB–ligand–receptor interaction tetramers weighted by the similarity (C‐scores) of the response in the two obesity groups versus control. The mean *p‐*values for the C‐scores of the corresponding genes were assigned to the tetramers, indicating the significance of the interaction as a response to maternal obesity. From the weighted CCC network, the in‐degree and out‐degree were calculated.

### 4.9 Overlapping With the GWAS and Placenta‐Specific QTL Associated Genes

We used the GWAS associated with birth weight (fetal GWAS) [82] and childhood BMI [83]. Furthermore, we examined placenta‐specific QTL linked with birth weight [84, 85]. Bhattacharya et al. reported placental genetically regulated expression (GReX) genes and only four GReX genes showed associations with fetal birth weight (*DUSP12*, *UBA3*, *FAM114A1*, *CMTM4*), although overall GReX did not explain significant trait heritability [84]. Tekola‐Ayele et al. identified 29 protein‐coding genes with significant eQTL–eGene associations with birth weight. To map the variants to genes, we used MAGMA to analyze the statistical association between the aggregate genetic risk at the gene level [86]. To ensure reliable results, we excluded cell types from this analysis with fewer than 10 DEGs in our data. The DEGs from the remaining placental cell types were overlapped with the GWAS or QTL associated genes aforementioned.

### 4.10 Adipose Spheroid Establishment

Adipose spheroid (AS) obesity conditions were established as previously described [77]. Briefly, cryopreserved human primary stromal vascular fraction of white adipose tissue of female origin was seeded in culture flasks with DMEM/F‐12 Glutamax, 10% FBS, and 1% penicillin‐streptomycin. After reaching the confluency of around 70%, cells were trypsinized and seeded into ultra‐low attachment 96‐well plates (Corning) with 5000 cells per well and centrifuged at 150 g for 2 min. After 4 days, the culture medium was changed to serum‐free differentiation medium (William's E supplemented with 2 mm L‐Glutamine, 100 units/mL penicillin, 100 µg/mL streptomycin, 10 µg/mL insulin, 10 µg/mL transferrin, 6.7 ng/mL sodium selenite and 100 nm dexamethasone; *differentiation cocktail*, 500 µm 3‐isobutyl‐1‐methylxanthine (IBMX), 10 nm hydrocortisone, 2 nm 3,3’,5‐Triiodo‐L‐thyronine, 10 µm rosiglitazone, 33 µm biotin and 17 µm pantothenic acid, and *free fatty acids (FFAs)*, 160 µm oleic acid; 160 µM palmitic acid conjugated to 10%BSA at a molar ratio of 1:5). After 48 h, half of the medium was changed and subsequently every 3–4 days for 17 days. After differentiation, the spheroids were transferred into the maintenance media supplemented with FFAs for four days before the chip co‐culture, and the media were exchanged every 2 days to phase out the differentiation growth factors.

### 4.11 Trophoblast Organoid Culture Establishment

The Swedish ethical review authority (Nr. 2023‐01276‐01) reviewed and approved the protocol. Villous trophoblast primary cells were isolated from human placental tissue as described previously [79]. Briefly, the isolated villi were fragmented and washed intensely. Then, the tissue was digested with 0.2% trypsin (Alfa Aesar, J63993‐09)/0.02% EDTA (Sigma‐Aldrich, E9884) and 1 mg/mL collagenase V (Sigma, C9263), and further disrupted by pipetting up and down with a serological 10 mL pipette, around 10–15 times. The cellular digests were combined and washed with Advanced DMEM/F12 medium (Life Technologies 12634‐010), and cells were counted with an automatic cell counter (TC20 BioRad). After, the cells were pelleted and resuspended in pre‐thawed Matrigel (Corning 35 623). Domes of Matrigel/cells (20 µL) were seeded in a 48‐well culture plate and placed in an incubator at 37°C with 5% CO_2_ for 3 min, then the plate was turned upside down so that cells distribute evenly within the Matrigel domes. The domes were incubated for a total of 15 min before supplemented with 250 µL of trophoblast organoid maintenance medium, composed with: Advanced DMEM/ F12 (Life Technologies, 12634‐010); 1× B27 (Life Technologies, 17504‐044); 1× N2 (Life Technologies, 17502‐048); 10% FBS (Cytiva HyClone, SH30070.03); 2 mm L‐glutamine (Life Technologies, 35050‐061); 100 µg/mL Primocin (InvivoGen, antpm‐1); 1,25 mm NAC (SigmaAldrich, A9165), 500 nm A83‐01 (Tocris, 2939); 1,5 µm CHIR99021 (Tocris, 4423); 50 ng/mL hEGF (Gibco, PHG0314); 80 g/mL human R‐spondin1 (R&D systems, 4645‐RS‐100); 100 ng/mL hFGF2 (Peprotech, 100‐18C); 50 ng/mL hHGF (Peprotech, 100‐39); 10 mm nicotinamide (Sigma‐Aldrich, N0636‐100G); 5 µm Y‐27632 (Sigma, Y0503‐1MG); 2.5 µm prostaglandin E2 (PGE2, R&D systems, 22‐ 961‐0). Medium was replaced every 2‐3 days. hCG was measured to ensure proliferation of trophoblast cells using professional pregnancy test strips (Gravidetstest GI29100, Medistore). Small trophoblast organoids became noticeable, and positive hCG tests were obtained around 10‐15 days after establishment.

### 4.12 Maintenance and Recovery of Trophoblast Organoids

Passages were performed every 5–7 days, as previously described [79]. Each Matrigel dome was disintegrated by pipetting the medium up and down and by scraping. Organoid suspension was washed and digested in pre‐warmed StemPro Accutase (Gibco, A11105‐01) with Y‐27632 (Sigma, Y0503‐1MG) for 10 min at 37°C. After carefully removing the supernatant, 200 µL of Advanced DMEM/ F12 (Life Technologies, 12634‐010) was added before performing mechanical dissociation by at least 200 ups and downs, using an automatic pipette. Cell suspension was washed and resuspended in cold Matrigel and plated into 20 µL domes with the desired cell density. Trophoblast organoid medium (250 µL) was added, after 15 min of incubation at 37°C with 5% CO_2._

After 7 days of culture, TOs were removed from Matrigel. For that, 250 µL of Cell Recovery Solution (Corning 354 253) was added to each well. The plate was incubated on ice for 45 min. The Matrigel domes were dissociated by gently pipetting up and down, and the organoid/cell recovery solution was collected and centrifuged. After removing the supernatant, the organoid pellet was washed and resuspended in cold pre‐thawed Matrigel. Tubes were kept on ice until the assembly of the microfluidic co‐culture.

### 4.13 Co‐Culture of Trophoblast Organoids With Adipose Spheroids on a Microfluidic Device

To co‐culture obese AS with TOs, we used multilayered microfluidic devices made of off‐stoichiometric thiol–ene–epoxy (OSTE+), poly(methyl methacrylate), and polydimethylsiloxane [76]. The OSTE+ parts, which hold the tissue chambers (*D* = 6 mm) and microfluidic channels (*W* = 1000 µm, *H* = 500 µm), were produced from OSTEMER‐322 (Mercene labs, Sweden), as previously described. The polydimethylsiloxane layer was cast, and the poly(methyl methacrylate) layer was cut using a micromilling machine (Minitech Machinery Corp., USA). The AS were grown and differentiated for 17 days and washed in maintenance media before the chip culture. Between 20 to 30 AS were loaded in the first chambers of the co‐culture chip, and 17 µL domes of TOs (grown for seven days after splitting) were seeded in Matrigel in the second chambers of all devices. The control chips were only seeded with TOs. To mimic an obese environment, obesogenic media were perfused at a constant flow rate of 5 µL/minute using an NE‐1600 SyringeSIX Programmable Multichannel Syring Pump (New Era Pump Systems Inc.). After 4 days, TOs were collected for RNA extraction.

### 4.14 RNA Extraction and Sequencing of Trophoblast Organoids

TOs for RNAseq analysis were recovered from Matrigel using Cell Recovery Solution (Corning, 354 253), as described previously. Cold TRI reagent (500 µL; Sigma‐Aldrich) was added and incubated on ice for 5 min. Then 100 µL of chloroform was added, vortex mixed, and centrifuged. Around 200 µL of supernatant was recovered, and the same volume of isopropanol was then added. The mixture was further mixed and loaded on a Minicolumn (ReliaPrep RNA Miniprep, Promega). The next steps were performed following the manufacturer's instructions and included DNase treatment.

Prime‐seq protocol was used to prepare RNA sequencing libraries as previously described [113]. Briefly, samples with 10 ng/µL of extracted RNA were used for reverse transcription and pre‐amplification. The cDNA was quantified using the PicoGreen dsDNA Assay Kit (ThermoFisher Scientific) and qualified using the Bioanalyzer High Sensitivity DNA chip (Agilent), before subsequent library construction using the NEBNext Ultra II FS Kit (New England Biolabs). Then the libraries were sequenced on Illumina NovaSeq 6000 at Novogene, UK, at an average depth of 20 million reads per sample.

### 4.15 Downstream Analysis of Trophoblast Organoid RNA Sequencing

Quality of the raw data was checked using FastQC (version 0.11.9), trimmed of poly(A) tails using Cutadapt (version 4.1), before filtering using the zUMIs pipeline (version 2.9.7) [114]. The filtered data were then mapped to the human genome using STAR (version 2.7.10a) [115], and reads were counted using RSubread (version 2.12.0) [116]. Lowly expressed genes (count sum < 50 for all samples) were filtered out before differential analysis. Differential expression analysis on RNA sequencing data was done using the DESeq2 package (version 1.36.0). DEGs between adipose spheroid (*n *= 4) and medium control groups (*n *= 3) were defined as adjusted *p*‐value < 0.05 and fold‐change > 2 or <−2.

The TOs were composed of CTB and STB. Cell type deconvolution was performed using the CIBERSORT [81] method on TO RNA‐seq counts, with we used a public snRNA‐seq dataset of trophoblast organoids (GSE288650_STBin) [117]. The STB and CTB were evenly sampled at 6617 each. The counts were both normalized to TPM, and the S‐mode batch correction was applied before estimating the nuclei type composition. Group differences in cell type proportions were compared using the Mann–Whitney *U*‐test.

The overlaps of the DEGs in TOs in obesity‐like conditions with the genes that were commonly regulated in O‐A and O‐L in CTB and STB were tested using a hypergeometric test, with a greater than the alternative hypothesis for enrichment testing, and universe size being 10 681, the number of genes that were expressed in organoid samples (average count per million > 1).

### 4.16 Statistical Analysis

For clinical and phenotypic comparisons across groups (maternal age, height, BMI, birth weight, placental weight, and placental efficiency), data were analyzed using GraphPad Prism 9.4.1 (GraphPad Software, San Diego, CA, USA). The Kruskal–Wallis test followed by Dunn's multiple comparisons test was performed to compare across Control (*n *= 4), O‐A (*n *= 4), and O‐L (*n *= 4) groups. A *p*‐value < 0.05 was considered statistically significant. Data were presented as mean ± standard deviation unless otherwise specified.

## Author Contributions

H.J. contributed to the design of the work, analysis, and interpretation of data, the formulation of the C‐score used in this work, and drafted the work. E.D. contributed to the design of the work and the acquisition of the data. D.P. contributed to the acquisition of the trophoblast organoids data and revised the work. N.T. contributed to the acquisition of the adipose spheroids and microfluidic co‐culture system. R.Z.S. fabricated and characterized the microfluidic devices. M.O. contributed to the acquisition of the placental tissues. P.R.J. contributed to the acquisition of the trophoblast organoids and revised the work. A.Z. contributed to revising the work. C.L. contributed to the library construction of the placental tissues. M.A.M. contributed to the acquisition of the placental samples. E.S.‐V. contributed to the acquisition of the samples and revised the work. V.M.L. contributed to the microfluidic co‐culture system and revised the work. Q.D. conceptualized and designed the work, interpreted the data, and drafted the work. All authors contributed to revising the manuscript.

## Conflicts of Interest

V.M.L. is co‐founder, CEO, and shareholder of HepaPredict AB, as well as co‐founder and shareholder of Shanghai Hepo Biotechnology Ltd. The other authors declare no conflicts of interest.

## Supporting information




**Supporting File 1**: advs73868‐sup‐0001‐SuppMat.docx.

**Supporting File 2**: advs73868‐sup‐0002‐Supplementary_Table_1.xlsx.

**Supporting File 3**: advs73868‐sup‐0003‐Supplementary_Table_2.xlsx.

**Supporting File 4**: advs73868‐sup‐0003‐Supplementary_Table_3.xlsx.

**Supporting File 5**: advs73868‐sup‐0004‐Supplementary_Table_4.xlsx.

**Supporting File 6**: advs73868‐sup‐0005‐Supplementary_Table_5.xlsx.

**Supporting File 7**: advs73868‐sup‐0006‐Supplementary_Table_6.xlsx.

## Data Availability

Raw snRNA‐seq data from the placenta tissues and Raw RNA‐seq data from trophoblast organoids were deposited in EGAS50000000834. The explanation and script for C‐score calculation are available at https://denglab‐ki.github.io/cscore/, and archived in https://zenodo.org/records/18071799. The notebooks to reproduce the presented results are available at https://github.com/Denglab/obesity_paper. Source data for reproducing the figures are deposited at Zenodo https://doi.org/10.5281/zenodo.14549513. Data on birth weight has been contributed by the EGG Consortium using the UK Biobank Resource and has been downloaded from www.egg‐consortium.org, specifically: Birth Weight Summary Data—Fetal GWAS (2016), “Trans‐ancestry meta‐analysis of up to 153,781 individuals” dataset. Data on childhood body mass index have been contributed by the EGG Consortium and have been downloaded from www.egg‐consortium.org, specifically: Childhood BMI Summary Data (2020).
