# Supplementary material for: Decoding Human Placental Cellular and Molecular Responses to Obesity and Fetal Growth
Source: Adv Sci (Weinh). 2026 Jan 20;13(17):e09691. doi: 10.1002/advs.202509691 (PMC13042494; doi:10.1002/advs.202509691)
Supplement: Supplementary file 1 — Supporting File 1: advs73868‐sup‐0001‐SuppMat.docx. [file ADVS-13-e09691-s006.docx]

# Supplementary Figures

**
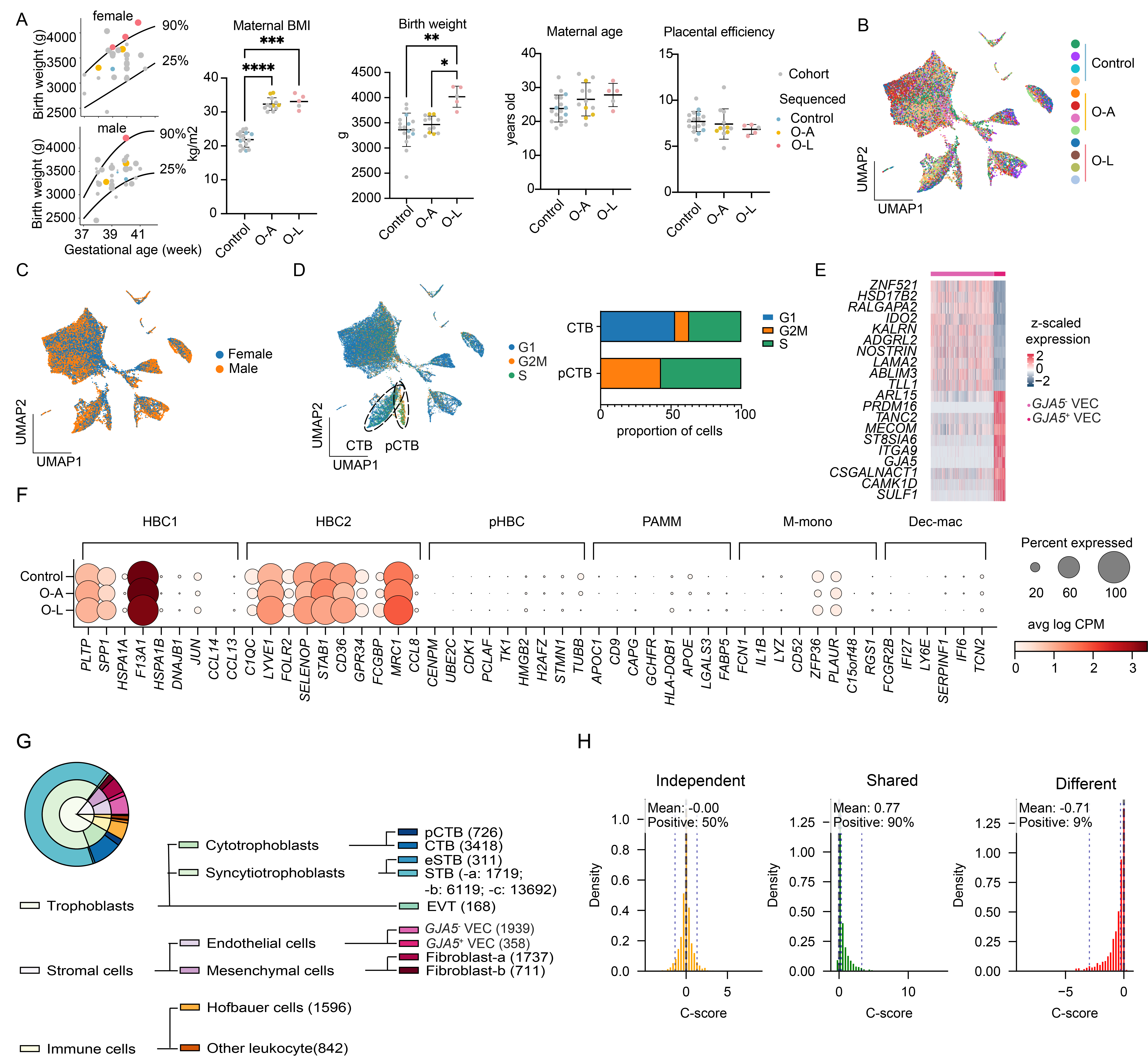
**

**Figure S1. snRNA-seq of the human placentas from normal-weighted controls and women with obesity. (A)** The birth weight and the gestational week for female and male fetuses in the cohort, which defined the appropriate-for-gestational-age (AGA) or large-for-gestational age (LGA) groups. And the comparison of maternal BMI, birth weight, maternal age, and placental efficiency between groups. The samples for snRNA-seq were colored by group. Sample size in the cohort: Control (n=18), O-A (n=12), O-L (n=5), using Kruskal-Wallis test followed by Dunn's multiple comparisons test. Error bars indicate mean±sd. (**B)** Two-dimensional Uniform manifold approximation and projection (UMAP) embedding of the nuclei colored by individual. (**C)** UMAP embedding of the nuclei colored by fetal sex. (**D)** UMAP embedding of the nuclei colored by the inferred cell cycle (left). The proportion of nuclei of CTB and pCTB in G1, G2M, or S phase of the cell cycle (right). (**E)** The heatmap shows the gene expression of the differentially expressed genes between the two types of VEC (GJA5+ n= 367, GJA5- n=2,113). The color bar indicates the mean z-scored gene expression of a gene. (**F)** Mean expression in Hofbauer cells captured in this study (dot color, gene expression scaled to unit variance) and fraction of expressing nuclei (dot size) of marker genes distinguishing term Hofbauer cells, placenta-associated maternal macrophages (PAMM), maternal monocyte (M-mono), Decidua macrophage (Dec-mac).^[117]^ (**G)** Pie plot showing the percentage of the major cell types and their hierarchy. The total cell type number of 12 samples was written beside the cell types. (**H)** Histograms show the distribution of C-scores for simulated gene expression contrasts (n = 2,000 genes). In independent experimental design: fold change follows normal distribution with mean=0, sd=1, and and the FDR of 20% of the genes are below 0.05; In common study design: fold change of the second comparison is the first added a random noise (noise level=0.3), and and the FDR of 20% of the genes are below 0.05; In divergent study design: fold change of the second comparison is the opposite to the first added a random noise (noise level=0.3), and the FDR of 20% of the genes are below 0.05. Controls: normal-weighted women (n=4); O-A: women with obesity and appropriate for gestational age baby; O-L: women with onesity and large for gestational age baby; CTB: cytotrophoblasts; VEC: vascular endothelial cells; EVT: extravillous trophoblasts; pCTB: proliferative CTB; STB: syncytiotrophoblasts; eSTB: early syncytiotrophoblasts.


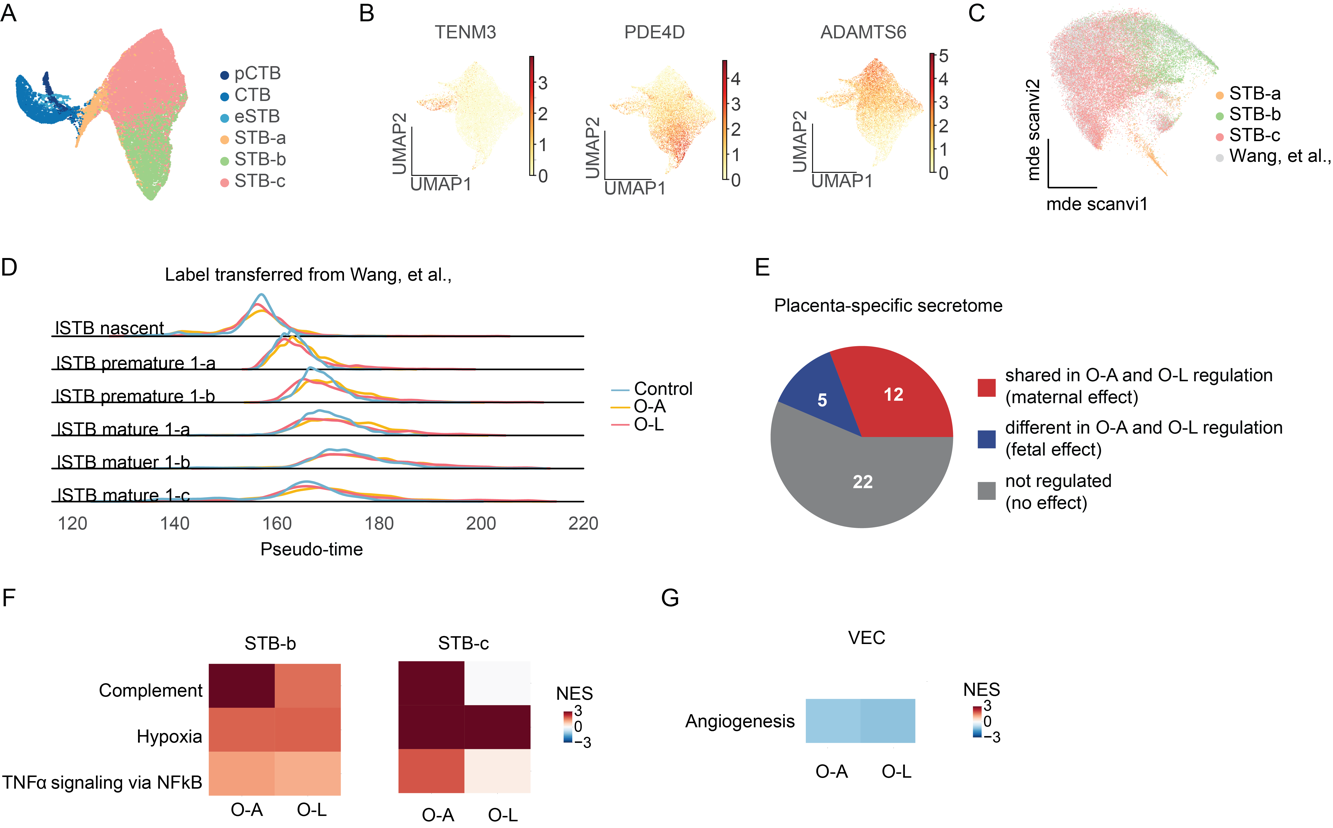


**Figure S2. The three states of syncytiotrophoblast nuclei and their transcriptional response to maternal obesity.** (**A)** UMAP representation of trophoblasts colored by marker genes. (**B**) UMAP representation of trophoblasts colored by marker genes by cell types. (**C)** SCANVI^[39]^ integration of the nuclei in this study and Wang et al., colored by the labels annotated in this study. (**D)** Density plot of the nuclei from this study, and the labels were transferred from Wang et al., after integration with the STB nuclei from the term placentas that they sampled. **(E)** The proportion of shared or different DEGs in O-A and O-L among the placenta-specific secretome.^[47]^ **(F)** GSEA of hallmark pathways for STB-b and STB-c psudo-bulk data. **(G)** GSEA of hallmark pathways for VEC psudo-bulk data.

**
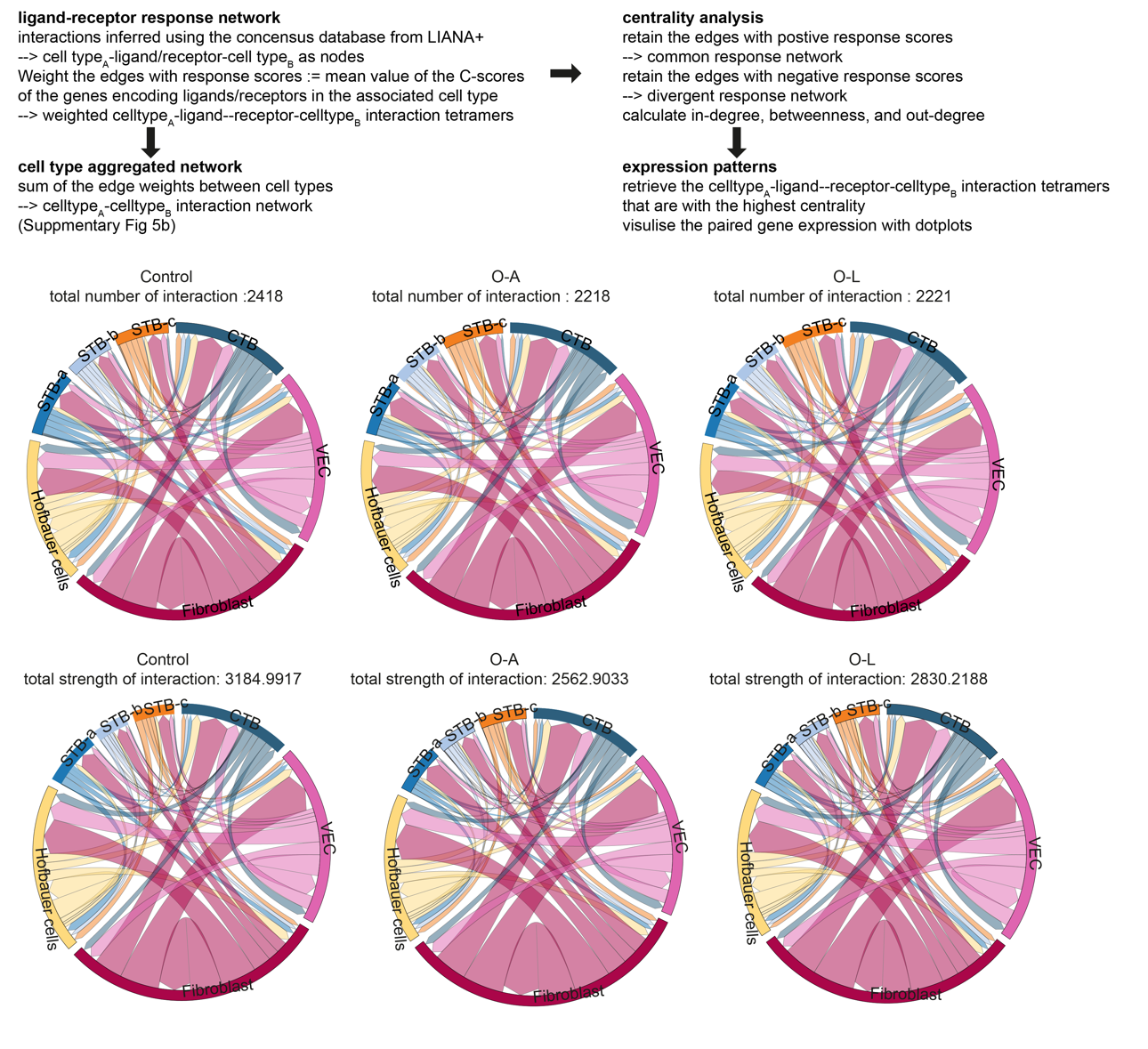
**

**Figure S3. Communicative pathways across cell types in human placental villi.**  Procedures of cell-cell communication network analysis (top panel). And a circos plot summarizing counts, and strength (weight) of ligand receptor pair between cell types generated by Liana+ ^[70]^ (bottom panel).
